# Comprehensive Review of Silver Nanoparticles in Food Packaging Applications

**DOI:** 10.3390/ijms26209842

**Published:** 2025-10-10

**Authors:** Erkan Efe Okur, Furkan Eker, Emir Akdaşçi, Mikhael Bechelany, Sercan Karav

**Affiliations:** 1 Department of Molecular Biology and Genetics, Çanakkale Onsekiz Mart University, Çanakkale 17100, Türkiye; erkanefe.okur@gmail.com (E.E.O.); furkan.eker@stu.comu.edu.tr (F.E.); emirakdasci@stu.comu.edu.tr (E.A.); 2 European Institute for Membranes (IEM), CNRS, ENSCM, UMR 5635, University Montpellier, Place Eugène Bataillon, CEDEX 5, F-34095 Montpellier, France

**Keywords:** silver nanoparticles, antibacterial activity, antioxidant activity, agricultural applications, food packaging, food preservation, toxicity mechanisms

## Abstract

In recent years, the use of silver nanoparticles (AgNPs) in various fields has been investigated due to their highly potent properties. One of these areas is the adaptation of AgNPs to food packaging/preservation technologies. The primary reasons for the use of AgNPs in food preservation studies are their high levels of antibacterial, antioxidant, and antifungal activities. In particular, the slow and controlled release of silver provides a sustained protective effect throughout the contact period of AgNP-integrated packaging with food and reduces microbial load by preventing biofilm formation. Furthermore, high thermal stability of AgNPs provides high protection to foods under various conditions. Their high surface-area-to-volume ratio, making them effective even at low concentrations, further supports AgNPs as a promising alternative in food preservation technologies. Moreover, their ease of surface modification facilitates the integration of these nanoparticles (NPs) into polymer matrices, biodegradable films, and coatings. Additionally, some AgNP-based films are also used in smart packaging applications, providing a color change indicator of degradation. Their broad pH tolerance enhances their applicability to a variety of food types, from dairy to meat products. For all these reasons, AgNPs are considered as one of the essential components of innovative food packaging to slow down food spoilage, prevent microbial contamination, and provide safer, longer-lasting products to the consumer, and studies on them are ongoing.

## 1. Introduction

Food safety and shelf-life extension have become globally important issues today due to growing populations, complex supply chains, and increased awareness of foodborne illnesses [1]. Contamination by microorganisms is the leading cause of food spoilage and oxidation, moisture imbalance, and inadequate packaging protection lead to reduction in quality and waste [2]. Traditional packaging often offers only physical protection; it lacks microbial inhibition or spoilage retardation. Therefore, research into active and smart packaging systems to both enhance food safety and extend shelf life is rapidly increasing [3]. In this context, active packaging modifies the food microenvironment by adding ingredients such as deoxidizers, antimicrobials, or ethylene inhibitors, directly preventing spoilage and extending shelf life. Smart packaging, on the other hand, uses sensors, indicators, or data carriers to monitor the safety and quality of food products in real time, providing valuable information to producers and consumers. Especially, color change indicators, the most commonly used of the previously discussed applications, are smart packaging components that visually indicate environmental changes through color change using dyes (most commonly synthetic pH dyes or natural pigments such as anthocyanin, betalin, and curcumin) sensitive to pH, gases (such as CO_2_, volatile amines), or microbial metabolites produced during the food spoilage process. These indicators are commonly used on meat, fish, poultry, dairy, fruit, vegetables, and bakery products, and serve as practical tools for monitoring freshness, detecting spoilage, and ensuring overall quality during storage and distribution [4]. Different food models, such as pork, shrimp, and silver carp, offer important alternatives for researchers in smart packaging applications based on color change. The anthocyanin-enriched gelatin films or AgNP–biopolymer composites used in these examples provide distinct and gradual color transitions that directly reflect freshness, allowing not only product safety monitoring but also rapid, visual feedback to the consumer. This clearly demonstrates the versatility and practical value of smart packaging systems for real-world applications [5,6,7,8]. Thus, while active packaging represents an interventionist strategy aimed at protection, smart packaging focuses primarily on observation and monitoring; together, they integrate both protection and real-time feedback, creating a safer and more transparent food supply chain [9].

AgNPs are a class of nanomaterials characterized by dimensions typically ranging from 1 to 100 nanometers. Compared to their bulk counterparts, they exhibit enhanced surface-area-to-volume ratios, which contribute to their superior physicochemical reactivity and functional capabilities [10]. AgNPs can be used in many different areas thanks to their numerous properties such as antioxidant, antibacterial, and anticancer activities [11]. One of these areas is the use of AgNPs in various food packaging and preservation applications, thanks to their high antioxidant and antibacterial properties [12]. These antibacterial properties occur through mechanisms such as membrane damage, reactive oxygen species (ROS) production, and DNA/protein degradation. Furthermore, AgNPs can be modified using various methods to enhance their biological effects [13].

The synthesis process of AgNPs has great importance for acquiring specific physical and chemical properties that determine their application area. Depending on the reducing agents used at this stage, the properties of the resulting AgNPs are tailored to meet the requirements of the intended applications [14]. AgNPs can be synthesized through two different approaches: top-down and bottom-up. The top-down methods involve only physical synthesis techniques, whereas bottom-up methods include both chemical and biological synthesis techniques. Physical and chemical synthesis techniques are fast, and much cheaper than biological synthesis but they are also not biodegradable compared to biological synthesis methods. There is growing interest in the green synthesis approach, which offers several advantages specific to food packaging applications [15].

AgNPs can be widely used in nanocomposite films to increase antimicrobial and oxidative stability in food packaging applications [16]. AgNPs show broad-spectrum activity against both Gram-positive and Gram-negative bacteria, exhibiting potential to extend the shelf life of foods and prevent spoilage [17]. When integrated into packaging films, AgNPs are released from the film surface in a controlled manner, damaging the cell membranes of microorganisms and disrupting proteins and DNA. However, the use of appropriate reducing agents is important for the stable and functional integration of AgNPs into the films, as they initiate the reduction in silver ions (Ag^+^) to Ag^0^ and form AgNPs [18]. It also determines particle size, shape, and distribution; these properties have a direct impact on the antimicrobial effect of AgNPs [19]. For the food packaging applications, choosing biocompatible and non-toxic reducing agents is essential for both health and environmental sustainability. Therefore, the reducing agent used in AgNP synthesis plays a critical role in both efficacy and film performance. Therefore, the selection of the reducing agent is of great importance not only in terms of environmental safety but also in determining the properties of the obtained NPs.

**Figure 1:**
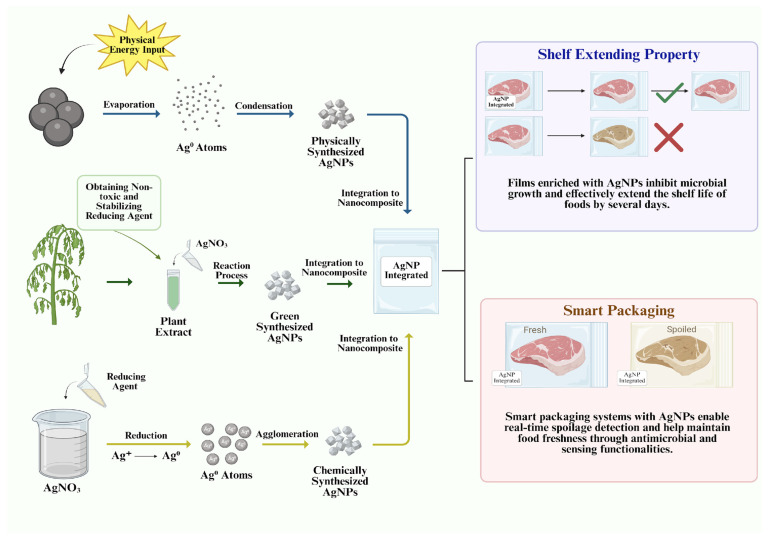
The synthesis methods of AgNPs and their functional integration into active and smart packaging systems [23].

Building on these advantages, the unique physicochemical characteristics of AgNPs, particularly their small size and high surface-area-to-volume ratio and their synthesis methods, further enhance their effectiveness in active and smart food packaging applications by providing specific mechanical and chemical properties [20]. These aspects are comprehensively illustrated in (Figure 1). Currently, various types of NPs are being investigated (AgNPs, gold NPs, protein-based NPs, polymeric NPs, iron oxide NPs, etc.) and AgNPs stand out with their antioxidant and antibacterial properties, positioning them as promising alternatives to conventional packaging methods [21,22].

This review provides a comprehensive overview of AgNPs as emerging agents in food preservation, beginning with their fundamental properties and underlying antibacterial and antioxidant mechanisms. It compiles recent progress on AgNP-integrated packaging materials, highlighting real-world applications and performance in extending the shelf life of diverse food products. In addition, particular attention is given to potential toxicological risks, migration behavior, and the ongoing debates surrounding consumer safety and regulatory considerations. By bringing together these perspectives, the review aims to offer a balanced and critical understanding of AgNPs in food preservation, bridging innovative technological advances with practical and safety-related challenges.

## 2. Protective Properties of AgNPs: Antibacterial and Antioxidant Activities

Due to their antibacterial and antioxidant properties, the use of AgNPs in food packaging applications has been a subject of considerable research in recent years. Although the antibacterial activity of AgNPs is generally mediated by the production of ROS, they can also exhibit antioxidant properties based on preventing the formation of these radicals or neutralizing them. These antibacterial and antioxidant properties can be manipulated by the different physicochemical properties of AgNPs such as size, shape, and surface chemistry.

### 2.1. Antibacterial Properties

AgNPs have been among the most studied and debated nanomaterials of the past decade, largely due to their immense antibacterial properties. Recent studies have highlighted their effectiveness against various bacterial strains, including multidrug-resistant (MDR) pathogens. Studies have shown that AgNPs exhibit significant bactericidal activity against both Gram-positive and Gram-negative bacteria [24]. Moreover, the AgNPs can also inhibit biofilm formation by disrupting bacterial signaling pathways and limiting the production of essential biofilm components. Consequently, they are known for their potent bactericidal activity against several foodborne pathogens, highlighting their potential in agricultural applications, especially in food preservation studies [25].

#### 2.1.1. Mechanism Insight

The first observation of the antibacterial properties of AgNPs was due to the accumulation of these particles in the membranes of bacteria, where their presence in the same environment induced membrane aggregation and structural disruption [26,27]. This property of AgNPs is known to be due to the ability of Ag^+^ to bind quite easily to amines, phosphates, and thiols. These functional groups play key biological roles, appearing in the side chains of amino acids, the backbone of nucleic acids, and the active sites of enzymes. Therefore, they potentially interfere with vital cellular functions and contribute to the antimicrobial properties of AgNPs. Given its low selectivity, Ag^+^ is assumed to promote bacterial killing by interacting with several targets simultaneously [28].

In recent studies, the main mechanisms of the antibacterial properties of AgNPs are categorized under various main pathways: production of ROS, disruption of the cell membrane, and interference with cellular DNA and protein content [29,30].

ROS are oxygen-derived molecules that are more reactive than oxygen (O_2_) and include both radical and non-radical types. They have been produced in almost all living cells. ROS can be generated in different parts of the cell, such as the chloroplasts, mitochondria, plasma membrane, peroxisomes, apoplast, endoplasmic reticulum, and cell wall [31,32]. ROS have attracted attention due to their emergence as a result of some cellular events, enzymatic activities, extracellular interactions, and their contribution to the formation of antibacterial properties. They disrupt the structural and functional integrity of bacteria by oxidative damage to cell membranes, proteins, and nucleic acids. Moreover, oxidative stress (OS), as seen in cases where ROS induction is excessive, is a key factor for the antibacterial effect of ROS [33]. However, uncontrolled increase in the level of ROS can lead to damage to host cells and cause toxicity, which will be evaluated on further sections. One of the most important features of AgNPs is their ability to induce excessive levels of ROS production, which accumulate in bacterial DNA, proteins, and membranes, leading to OS and disrupting essential cellular functions [11]. The antibacterial effect of ROS occurs as a result of three different mechanisms that damage the living cell in different ways. One of these mechanisms is lipid peroxidation, in other words, damage to the lipid-containing carbon–carbon double bonds and initiating a chain reaction that disrupts membrane integrity and permeability. Another mechanism is oxidative damage to the DNA, which can lead to single- or double-strand breaks [34]. The last mechanism is referred to as protein oxidation. Specifically, ROS bind to the hydrophobic moieties of amino acids, breaking peptide bonds and preventing proteins from performing their functions, as well as causing protein damage through carbonylation [35].

The high surface-area-to-volume ratio of AgNPs can lead to both the penetration of the inside of the cell or disruption of membrane integrity, increasing its permeability and causing pore formation in the cell wall. This phenomenon leads to a decrease in cell content. This large porous structure facilitates the accumulation of AgNPs inside the cell, allowing these NPs to interact directly with intracellular components. As a result, AgNPs can bind to critical molecules such as DNA and proteins, leading to their impairment, functional inhibition, and ultimately disruption of vital processes such as replication, transcription, and enzymatic activity. AgNPs can cause these deformations through binding with sulfur and phosphorus-containing proteins [36].

To sum up, there are different mechanisms that can contribute to antibacterial activities of AgNPs. One of them induces excessive ROS generation and causes the occurrence of inhibition in significant vital activities. The other two bind to cell membrane proteins, intracellular proteins, or DNA, causing disruption of these molecules. Certain physicochemical properties of AgNPs, such as size, shape, and surface charge, are highly influential in the mechanisms and level of potency; hence, these factors should be considered when discussing antibacterial activity.

#### 2.1.2. Factors Affecting Antibacterial Activity of AgNPs

##### Size

As previously stated, the physicochemical properties of AgNPs are extremely important in their antibacterial activity, with particle size being one of the important factors in determining their efficiency [37]. Also, the studies on toxic effects of AgNPs are highlighting that there is significant increase in the cytotoxic effects of AgNPs as the NP size decreases [38]. The effect of size on the bactericidal effect of AgNPs is known to arise from the inhibition of enzymatic activity and biosynthesis of bacteria. Studies have shown that smaller size NPs are both more toxic and more likely to inhibit these mechanisms than larger size NPs [39].

The high resistance of biofilm structures against antibacterial agents causes significant difficulties in treatment processes. While the efficacy of AgNPs for biofilm inhibition stands out as a remarkable alternative, the level of this efficacy largely depends on the particle size. Smaller AgNPs, thanks to their high surface-area-to-volume ratio, diffuse more effectively into the biofilm matrix and increase the antimicrobial effect by providing a larger contact surface with bacterial cells. In addition, since small particles have a higher capacity to trigger ROS production, they can have a more destructive effect on cell membrane integrity and metabolic functioning. Studies conducted in this direction have revealed that as the particle size decreases, a significant increase in the inhibition of biofilm formation is observed [28].

The antibacterial efficacy of AgNPs exhibited a clear size-dependent result, as evidenced by the minimum inhibitory concentration (MIC) values against* Escherichia coli* (*E. coli*) and *Staphylococcus aureus* (*S. aureus*). Notably, 7 nm AgNPs showed the strongest antibacterial activity with the lowest MIC values (6.25 µg/mL for *E. coli* and 7.5 µg/mL for *S. aureus*), compared to larger counterparts. In contrast, 89 nm AgNPs demonstrated significantly reduced efficacy, particularly against *S. aureus* (MIC: 33.71 µg/mL). These findings highlight that size is an important factor for antibacterial activity of AgNPs [40].

In addition, smaller-sized AgNPs synthesized from different organs of the Carduus crispus plant (especially AgNP-F obtained from the flower, ~22.5–33 nm) were found to exhibit stronger antibacterial activity against both Gram-positive and Gram-negative bacteria. The authors highlight that the particle size could be manipulated with the chosen plant organ and their activity was directly dependent on the size [36].

##### Shape

The shape of AgNPs depends on the synthesis methods used during their production. It is shown that in different studies, various shapes can demonstrate different levels of antibacterial activity and use of application. These shapes can be nanoplates, rod-shapes, spherical, nanoprisms, truncated triangular, and so on [41].

One of the studies exhibited noteworthy results that compare silver nanocubes (AgNCs) and silver nano-spheres (AgNSs) according to their antibacterial properties. Researchers observed that AgNCs have better antibacterial activity but exhibited the same level of cytotoxicity with AgNSs [42]. Another study discussed how shape affects the antimicrobial effect of AgNPs. In this study, the disk diffusion method was used to test differently shaped AgNPs and their antimicrobial effects. Researchers observed that spherical AgNPs showed the strongest antibacterial effect as they release more Ag^+^ ions due to their higher surface area than disk-shaped and triangular sheet-shaped AgNPs, respectively [43].

##### Surface Chemistry

The surface charge is another important physicochemical property for AgNPs. Similar to size and shape, surface charge can also be manipulated to confer different properties under various conditions. The resulting surface alterations affect both stability and interaction with other molecules depending on the specific modifications made [44]. These changes greatly influence the antibacterial and toxic properties of NPs.

In a study investigating the relationship between the antibacterial activity of AgNPs and their surface properties, it was seen that ultrasmall AgNPs modified with chitosan (CS) to obtain particles with positive charges on their surfaces showed significantly higher antibacterial and antibiofilm activity than negatively charged AgNPs of the same size [45]. Additionally, reactive groups present in the surface of AgNPs take part in the generation of ROS, which is thought to affect the antibacterial activity of AgNPs.

### 2.2. Antibiofilm Properties

AgNPs exhibit potent antibiofilm properties through a combination of physicochemical and molecular mechanisms. They can damage bacterial membranes, induce oxidative stress via silver ion release and ROS generation, and penetrate cells to trigger toxicity, all of which lead to cell lysis and inhibition of biofilm maturation [46]. Beyond ion release, AgNPs stably incorporated into material matrices such as glass ionomer cements effectively suppress bacterial adhesion, achieving stronger antibiofilm effects with lower silver content compared to conventional systems [47]. Synergistic strategies further enhance these effects. Silver–antibiotic combinations increase bacterial membrane permeability, facilitating antibiotic entry and boosting antibiofilm activity even at reduced dosages [48]. Similarly, AgNPs coupled with phytochemicals or antimicrobial peptides disrupt biofilm biomass and structure while downregulating key biofilm-related genes such as fimH in *E. coli* and lasI/rhII in *P. aeruginosa* [49,50]. Furthermore, recent studies highlight that AgNP-based coatings offer sustainable strategies to control microbial contamination not only by preventing bacterial adhesion but also by reducing the persistence of pathogenic biofilms on surfaces [51]. It is also known that AgNP-based nanocomposites can break down the cell wall and inhibit metabolic processes in addition to their ability to generate ROS, which is significant for antibiofilm activity, highlighting their applicability in food preservation technologies [52,53]. It has also been observed that AgNPs exhibit effective antibiofilm activity against drug-resistant bacteria [52]. Collectively, these findings highlight that AgNPs prevent biofilm formation through a multifaceted approach involving structural disruption, oxidative stress induction, and modulation of bacterial gene expression.

### 2.3. Antioxidant Properties

Beyond their antibacterial capabilities, AgNPs are also known for their high antioxidant activity. External antioxidants mitigate oxidative stress which can lead to various diseases when the natural antioxidants are not enough to inhibit excess radicals in the living cells. The ingestion of antioxidant-containing foods is necessary to prevent various diseases [54]. In this context, AgNPs have shown promising antioxidant potential and may serve as effective agents in food packaging and related applications aimed at managing oxidative stress.

One of the key mechanisms that constitute their potential is their ability to inhibit radicals through electron transfer processes, allowing AgNPs to donate and accept electrons. As a result, AgNPs can effectively stabilize ROS and inhibit oxidative chain reactions. This behavior is related to redox potential of silver, especially Ag^+^ and Ag^2+^ depending on the reaction conditions [55]. In the context of green synthesis, AgNPs are typically coated and stabilized with plant- or microorganism-derived chemicals such as enzymes, flavonoids, benzoic acids and cinnamic acids, strengthening the NP stability and ROS scavenging. Considering the influence of physicochemical properties of AgNPs on their antioxidant capability, tailoring synthesis parameters is essential to optimize their morphological and surface characteristics for enhanced radical scavenging efficiency. Together, these properties enhance the reducing power and free radical neutralization ability of AgNPs, which has been observed in in vitro antioxidant assays, including 2,2-diphenyl-1-picrylhydrazyl (DPPH) and 2,2′-azino-bis-3-ethylbenzothiazoline-6-sulfonic acid (ABTS) assays [55,56]. This allows AgNPs to exhibit an antioxidant effect directly by scavenging ROS, and also to indirectly influence antioxidant defense by modulating cellular ROS production. This creates a complex balance that varies with AgNP concentration and cell type.

To investigate the relationship between the physicochemical properties of AgNPs and their antioxidant activity, further research is necessary. In one particular study, researchers investigated how the size of AgNPs affect antioxidant activity. The Superoxide Dismutase (SOD) inhibitory activity test demonstrated a significant increase in SOD, Catalase (CAT) and Metallothionein levels during the first 24 h of exposure. In addition, an increase in the intensity of the 638 cm^−1^ and 1572 cm^−1^ bands in the Raman spectrum was detected and associated with increased antioxidant protein synthesis of the cells. However, especially after 72 h of prolonged exposure, it was observed that the antioxidant effect of AgNPs decreased and the toxic effect increased significantly. This result suggests that smaller-sized AgNPs (35 nm) are more effective antioxidant agents than larger-sized AgNPs (50 nm) [57]. In another study, the antioxidant activity of differently shaped AgNPs was compared. Spherical, hexagonal, and triangular AgNPs were tested for antioxidant activity using the DPPH assay. The results showed that particle shape had no significant impact on antioxidant activity, indicating that shape does not strongly influence this property [58].

**Figure 2:**
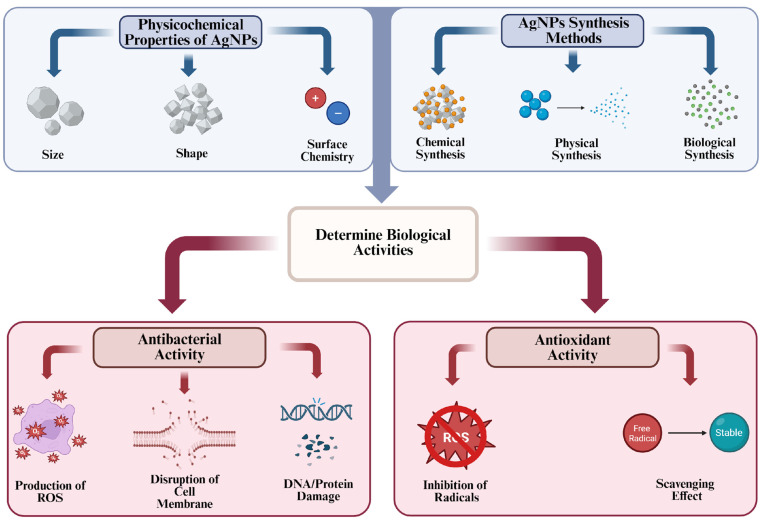
The biological behavior of AgNPs shaped by their physicochemical features and synthesis methods [59,60].

In general, studies show that AgNPs are useful materials for food packaging due to their antibacterial and antioxidant properties. In addition, the physical and chemical properties of these AgNPs, such as size and shape, can be easily modified by synthesis methods. This modification can further enhance the AgNP application to stop the growth of harmful microorganisms and protect food against oxidative degradation (Figure 2). Due to these properties, AgNPs are a good alternative for the development of composite materials that will keep food fresh and safe for longer.

## 3. Food Packaging Applications of AgNPs in Various Food Types

AgNPs have recently become very popular among researchers due to their antibacterial and antioxidant properties as well as their manageable properties such as size, shape, and surface properties. Although there are a variety of studies on the use of AgNP in food packaging technologies, it is a known fact that there is still a necessity for diverse studies regarding developing AgNP-based food packaging strategies. During these studies, various mechanical parameters are tested to determine the effectiveness of the developed NP-based packaging materials.

**Figure 3:**
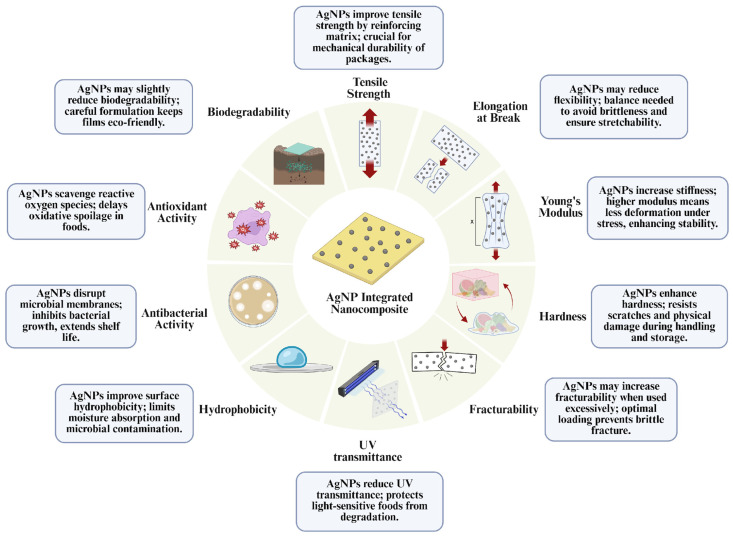
Illustration of the influence of AgNPs on the mechanical properties of the films through integration [65,66].

Tensile strength defines the maximum tensile force that edible films can withstand before breaking, while elongation at break refers to the percentage of stretching potential a material can achieve before breaking [61]. On the other hand, Young’s modulus is a parameter that points out resistance of the material to elastic damage and it is commonly used, particularly in nanocomposite production [62]. Similarly, hardness refers to the packaging film’s physical resistance to applied forces, and harder structures are more advantageous in resisting impact during shipping and storage. Fracturability refers to the film’s susceptibility to cracking and breaking and is particularly important for protecting fragile foods. UV transmittance indicates the film’s ability to transmit ultraviolet light; the lower this value, the greater the film’s protection against photochemical damage [63]. Similarly, visible light transmittance indicates the transparency level of food packaging materials. High light transmittance allows consumers to easily observe the product, while low transmittance can reduce light-induced degradation and help extend shelf life [64]. These mechanical properties of the mentioned AgNP-integrated films are mentioned in (Figure 3).

Furthermore, it has been shown that these properties can be enhanced by the addition of AgNPs in various applications. The use of AgNPs in packaging applications is becoming widespread and is currently under research. These comprehensive evaluations demonstrate the suitability of packaging for shelf life and food safety beyond microbial control. They have been widely applied in food packaging for products such as fruits, vegetables, meat, dairy, and baked goods, owing to their broad-spectrum antibacterial activity and antioxidant properties. When trying these preservation applications on foods, methods such as paper dipping, dipping, brushing, wrapping, and spray coating can be used [67,68,69]. In recent years, numerous studies have further explored their potential and applications. The studies and methods discussed in this section have been included to comprehensively evaluate various food types in this field.

### 3.1. Fruit

Shuying Li et al. impregnated AgNPs into pectin/gelatin composite. To synthesize AgNPs, they used tannic acid (TA/T) and AgNO_3_ and mixed with a solution that contains gelatin (G), pectin (P), and glycerol. Transmission Electron Microscopy (TEM) results showed that synthesized AgNPs have cubic structure and size ~23 nm. Seven different test groups that contain different concentrations of AgNPs were created. While the control group contained only pectin and gelatin, the remaining groups contained pectin, gelatin, TA, and AgNPs, and the test groups contained AgNPs at concentrations of 0.05, 0.5, 2.5, and 6.25, respectively. The most important aspect that distinguishes this study from other studies is the UV-blocking property of the film. A significant increase in UV transmittance was observed after the addition of TA and AgNPs to the film. It was also observed that the mechanical properties changed with the addition of TA and AgNPs. While a significant decrease in tensile strength and elongation at break (EAB) was observed with the addition of tannic acid to the P/G film, positive effects were observed at low concentrations of AgNPs and a decrease in tensile strength and EAB values was observed again at high concentrations. Antioxidant activity assays such as DPPH and ABTS have been applied to the films. While there is no antioxidant effect of P/G films, the addition of tannic acid showed notable antioxidant effects. Finally, the incorporation of AgNPs enhanced antioxidant activity at low concentrations; however, at higher concentrations, a minor reduction in antioxidant effect was observed. Moreover, the film that contained higher concentration of AgNPs showed the highest antibacterial effect among all test groups. TA showed antibacterial effects on S. aureus and E. coli as well, but not as effective as the AgNPs films. Furthermore, cytotoxicity tests also showed that films containing low concentrations of AgNP with tannic acid were not toxic, but conversely, films containing high concentrations of AgNP with tannic acid were classified as toxic. Finally, for the coating tests, strawberry has been used due to its short shelf life. Strawberries coated with polyethylene (PE) and the control group rotted but the groups that coated with P/G and P/G/T/AgNP0.5 showed no spoilage on the fourth day. However, on the 8th day, softening and mold were seen in the control group, PE group, and P/G group, while there was no bacterial growth or rotting in the strawberries covered by the P/G/T/AgNP0.5 film [70]. These results show that these films containing the correct concentration of AgNPs are an alternative method that can be effective in preserving the shelf life of foods with short shelf life and low post-harvest activity.

Another NP composite film produced by Zhao et al. was made using grape seed extract (GSE) to synthesize GSE-AgNPs. Characterization studies revealed that the sizes of the synthesized AgNPs were 23.8 nm and had spherical shape. Antifungal tests also applied to GSE-AgNPs and results showed that GSE-AgNPs had the most antifungal activity among other test groups such as GSE, PVP-AgNPs, and the control group containing only water. For the antioxidant assays, the DPPH method was applied and it was observed that the added GSE enhanced the antioxidant activity of AgNPs. In addition, PVP-AgNP and GSE-AgNP were compared and although it was observed that the antioxidant activity of both increased with increasing concentrations ranging between 6.25 μg/mL and 100 μg/mL, GSE-AgNPs were found to be more antioxidant than PVP-AgNPs. Finally, the shelf-life-enhancing effect of CS-GSE-AgNPs composite film on grapes was investigated. For this purpose, CS-GSE-AgNPs, CS-PVP-AgNPs, and just CS has been tested as a film on rotting grapes caused by fungus and observed for 5 days. Results showed the minimal mass loss observed in CS-GSE-AgNPs-coated grapes. Furthermore, while the decay percentage in CS-GSE-AgNPs was 45%, this value was 90% in the control group. Lastly, different tests such as titratable acidity, the sensory evaluation and total yeast and mold count (YMC) were applied and the composite with the most effective results was CS-GSE-AgNP [71]. As a result, the composite film developed in this study offers a novel perspective on food packaging and shelf-life extension applications.

Sharma et al. highlighted that AgNPs synthesized with bacteriocin, an antibacterial peptide, can be used to produce a novel food packaging material. For this purpose, bacteriocin has been produced via different prebiotic cultures such as *Lactobacillus pentosus* S6, *Lactobacillus crustorum* F11, and *Lactobacillus spicheri* G2. The activity values measured for purified* Lactobacillus pentosus* S6 are 8 × 10^3^ AU/mL, while the activity values measured for* Lactobacillus crustorum *F11 and *Lactobacillus spicheri *G2 are 6 × 10^3^ AU/mL and 8 × 10^3^ AU/mL, respectively. The well diffusion method has also been used to measure the antibacterial effect of bacteriocins. Inhibition zones of crude, partially purified and purified bacteriocins have been measured and since it was determined that the bacteriocin with the highest antibacterial activity was the purified ones, these bacteriocins were used in the rest of the study. AgNPs were characterized with Scanning Electron Microscopy (SEM) and TEM, showing a spherical shape, and F11-AgNPs, S6-AgNPs, and G2-AgNPs have 5 nm, 10 nm, and 20 nm radius, respectively. The produced AgNPs were tested in food packaging applications by dipping cellulose papers into these AgNP solutions. Blank paper and paper dipped only in AgNO_3_ were used as control groups. SEM was used again but this time to measure dispersion of AgNPs in the paper. SEM results determined that bacteriocin-AgNPs showed dense and uniform distribution but in paper coated with just AgNO_3_, this dispersion is unsteady. Moreover, antibacterial activity of these papers has been tested. Bacteria *S. aureus* and *Bacillus Cereus* (*B. cereus*) were used in the test because they appear to be prevalent in food spoilage. Five different test groups were determined such as S6-AgNP paper, F11-AgNP paper, G2-AgNP paper, paper with just AgNO_3_ (control 1) and finally blank paper (control 2). To observe antimicrobial effects of test groups, a spot-on-lawn method was used. According to results of incubation of these test groups, F11-AgNPs showed notable results as an antibacterial food packaging material. Although it was observed that all AgNP-coated papers showed high antibacterial effects on both bacteria, the one with the best antibacterial activity, i.e., the largest inhibition zone, was F11-AgNP. These results showed us that bacteriocin-synthesized AgNPs have strong antibacterial effect on food spoilage bacteria and this is an important indication that it is a promising food preservative material for food packaging. Given its potential, the shelf-life extension potential of this promising material also required investigation, leading researchers to conduct this study using tomatoes as a model system. The same five experimental groups used in previous tests were used in this real-life experiment. Each tomato was covered with one experimental group and these papers were pierced. Packages were stored at room temperature (18–20 °C) for 10 days. Different parameters such as appearance, firmness, and color change were measured. At the end of this 10-day experiment, the F11-AgNP-coated paper showed the best protection, while rot and mold were observed in tomatoes covered by the control groups. The G2- and S6-AgNP-coated papers appeared to be in better condition than the control groups, but softening and wrinkling were observed. Also, in color change experiments, F11-AgNP-coated paper preserves the color of the tomato best [72]. Taken together, the promising properties of AgNPs synthesized with bacteriocins synthesized from *Lactobacillus crustorum* F11 in innovative and green food packaging and shelf-life extension should be taken into consideration.

Another study aimed at extending the shelf life of fruits was conducted by Mao et al. The research team synthesized AgNPs (Ag@CS) using CS as a stabilizer, and UV-Vis analysis confirmed the successful formation of AgNPs. TEM analysis revealed average NP sizes ranging from 20 to 30 nm, and a homogeneous distribution was observed, confirming CS’s role as a stabilizer. *Bacillus velezensis* Q-84 was subsequently isolated from sweet cherries. Using a bacterium that protects fruit in the same fruit also increased the film’s compatibility with the plant species being protected. Lipoproteins (LPs) were then isolated from these bacteria. Analysis of the prepared lipopeptides was performed for LC-MS. Different concentrations of LP and Ag@CS NPs were tested and the most suitable concentrations for mixing the two were determined as LP: 0.64 mg/mL and Ag@CS NPs: 0.16 mg/mL. Subsequently, antimicrobial activity tests were performed on LP, Ag@CS NPs, and their mixture. These tests revealed that LP and Ag@CS NPs exhibited significant antibacterial activity against both Gram-negative (*E. coli*) and Gram-positive (*S. aureus*) bacteria. However, when used together, they exhibited the same level of antibacterial activity at lower doses. Next, the polyvinyl alcohol (PVA) solution was prepared and, after cooling to room temperature, glycerol was added and thoroughly mixed again. PVA/LP, PVA/Ag@CS NPs, and PVA/LP/Ag@CS NPs composites were prepared. These composites were also subjected to different characterization studies. Intermolecular interactions were analyzed using FT-IR. It was analyzed by SEM that the surface of the PVA/LP film was irregular, the PVA/Ag@CS NPs film had a smoother surface, and finally the PVA/LP/Ag@CS NPs film had the smoothest and densest surface. The highest TS and EAB values of the PVA/LP/Ag@CS NPs film were measured to be among the other films. Additionally, it was observed as a result of the analysis that PVA/Ag@CS NPs and PVA/LP/Ag@CS NPs films had the highest thermal stability. Additionally, water contact angle (WCA) tests were applied and it was observed that the highest hydrophobicity was in the PVA/LP/Ag@CS NPs film. The PVA/LP/Ag@CS NPs film also had the most effective value (53.5% reduction) in Water Vapor Transmission Rate (WVTR) tests. In ABTS tests performed to measure antioxidant effect, films containing LP were observed to have high antioxidant activity values. Finally, in antimicrobial activity tests, it was observed that the PVA/LP/Ag@CS NPs film had 90.2% inhibition effect on *E. coli* and 99.4% on *S. aureus*. Additionally, it was highlighted that it also had an inhibitory effect against molds and fungi (*Rhizopus stolonifera*: 68.5%, *Botrytis cinerea*: 65.7%). Real-life experiments were conducted to investigate the protective effects of different films on sweet cherries, namely on quality indicators such as deterioration, tissue degradation, and enzyme activity. Freshly harvested ‘Tieton’ cherries of similar size, undamaged, mature, but not over-softened samples were selected for the experiment. These samples were dipped in PVA, PVA/LP, PVA/Ag@CS NPs, and PVA/LP/Ag@CS NPs solutions, allowed to dry, and were analyzed for various parameters over 15 days. At the end of 15 days, the control group (no film application) showed a rotting rate of 87.2%, while this rate was 10% for PVA/LP/Ag@CS NPs-coated cherries. Additionally, firmness measurement was performed for all samples and the results showed that PVA/LP/Ag@CS NP-coated samples decreased the least. Moreover, polyphenol oxidase (PPO) and peroxidase (POD) activities were also measured and while PPO activity were lower in coated samples, POD activity first increased and then decreased in the groups containing LP [73]. In conclusion, the PVA/LP/Ag@CS NPs composite coating was the most effective solution in preventing both microbial deterioration and physiological softening. It significantly reduced the rotting rate and its effects on enzyme activities were in the direction of suppressing browning and preserving tissue strength. In addition, the comprehensive property of this study has greatly helped to draw attention to the high protective properties of the film produced.

Yang et al. also contributed to a study on the use of AgNPs in food preservation by synthesizing lichenan-AgNPs and performing a food preservation experiment on bananas with a CS-based matrix. L-AgNPs were synthesized by mixing lichenan (LC) and AgNO_3_ under optimal conditions, with LC acting as both a stabilizer and reducer. The particles were used to prepare CS-L-AgNP solutions, to which LC was added to form CS-LC-L-AgNP films. These films were cast and dried, and films containing three different AgNP concentrations were formed. These films were named CS-LAg1, CS-LAg-2, and CS-LAg-3 in order of AgNP concentration from smallest to largest (respectively, 66.5 mg/L Ag, 133 mg/L Ag, and 266.37 mg/L Ag). The synthesized L-AgNPs were subjected to characterization. TEM and SEM results revealed that the NPs were spherical/oval in structure and had a diameter of 6.4 nm. Energy-dispersive X-ray spectroscopy (EDX) analyses were also performed, and the Ag content was measured as 10.17%. Furthermore, X-ray diffraction (XRD) analyses revealed that the CS and CS-LC films were amorphous. Finally, the surfaces of the CS-LAg films were observed to be smooth and the AgNPs were homogeneously distributed via SEM. Atomic Force Microscopy (AFM) tests revealed that the films had low surface roughness. After that, mechanical properties of nanocomposite films were tested. While the TS value was measured as 15.06 MPa in the CS film, it was 35.97 MPa in the CS-LAg film, indicating that AgNPs caused a significant increase in the TS value. The YM value increased by 0.46 GPa in the AgNP-containing film and that means AgNP enhanced the hardness of the film. Researchers also measured the toughness and they found that the value of toughness of the film was 5.98 MJ/m^3^. As the last of the mechanical property tests, EAB was tested and a decrease in the EAB value of the AgNP added films was observed. Afterwards, optical properties were tested and although all films were observed to be transparent to certain levels, it was observed that the opacity level increased as the amount of AgNPs increased (the highest opacity was in CS-LAg3). Additionally, UVA and UVB transmittance were tested and CS-LAg film exhibited 95.47% and 98.95% protection, respectively. When measuring the barrier properties of the CS-LAg film, the moisture content was first examined, and it was found that the CS-LAg3 film (9.6%) contained significantly less moisture than the CS film (15.44%). Moisture absorption was also measured, and water uptake from the external environment was reduced in the CS-LAg3 film (17.56%) compared to the CS film (19.75%). Furthermore, the water vapor permeability (WVP) of the CS film was measured as 8.14 × 10^−11^, while that of the CS-LAg3 film was measured as 5.11 × 10^−11^. WCA values were also measured and it was found that the CS-LAg3 film was almost two times more hydrophobic than the pure CS film. In addition to other studies, carbon dioxide permeability was measured as 1.65 × 10^−7^ g/m·s in CS-LAg3 film. The oxygen permeability of the CS-LAg3 film was also determined as 1.96 × 10^−7^ g/m·s. This value indicates that the film has good gas barrier properties. Ag^+^ release tests were also performed, and although a rapid release was observed in the first 36 h, a slower and more controlled release was observed thereafter. Antioxidant activity of composite film and apple slice preservation were also measured. DPPH and ABTS analyses showed scavenging efficiencies of CS-LAg3 of 95.53% and 99.86%, respectively. It was also observed in cut apple samples that the browning and weight loss values in CS-LAg3-preserved apples were less compared to the control group apple samples. Photothermal conversion tests were also performed and it was concluded that CS-LAg3 can rapidly reach high temperatures and this feature triggers the antibacterial effect of the film. Tests for antibacterial activity showed that the antibacterial activity of the produced CS-LAg3 film against *E. coli* and *S. aureus* in the absence of Near Infrared (NIR) was 47.87% and 28.17%, respectively, while in the presence of NIR it was 99.69% and 99.11%. Finally, real-time tests were conducted. Banana, a fruit known to be prone to food spoilage, was chosen for further experiments. Bananas with no visible damage and similar shape were selected first. Four different groups were determined as control group, PE group, CS-LC group, and CS-LAg3 group. They were stored at 4 °C and for 6 days. Bananas were observed every 2 days by examining different parameters. After 6 days, darkening of the peel was observed in the control group, while the least browning was observed in apples protected with the CS-LAg3 film. After the PE film, the lowest weight loss was observed in bananas protected with the CS-LAg3 film (8.90%). Finally, firmness measurements were also conducted, and the results showed that the CS-LAg3 film slowed down banana softening the most [74]. To summarize, the photo-responsive performance and comprehensive characterization of the films distinguish this study from other studies.

Kowsalya et al. also produced antimicrobial food packaging material by integrating AgNPs synthesized from biological waste cassava shells into gelatin-based biodegradable films and characterized this film through various tests. First, AgNPs were synthesized from cassava shells and then pH optimized. The surface plasmon resonance (SPR) absorbance peak showed that pH = 7 was the optimal pH. TEM tests revealed that the formed AgNPs were spherical in shape and ranged from 10 to 45 nm in size. Phytochemical tests revealed compounds in cassava shells that reduce and stabilize Ag^+^ ions, such as carbohydrates, tannins, and saponins. The antimicrobial properties of the synthesized AgNPs were then tested. *Bacillus subtilis*, *S. aureus*, *Pseudomonas aeruginosa* (*P. aeruginosa*), *and E. coli* were used as bacteria, while *Aspergillus *sp. was used as fungi. Well diffusion results showed the highest inhibition against *S. aureus*, while milder inhibition against fungi was observed. Next, gelatin–AgNP film was prepared. A control film containing no AgNPs was produced. FESEM analyses revealed a uniform and homogeneous distribution of AgNPs within the gelatin. Finally, applications were conducted on sapodilla fruit to test the shelf-life-extending effect of AgNP-containing gelatin films. In fruits examined at room temperature for 12 days, the control group shriveled, starting on day 3, while the gelatin–AgNP-coated fruits maintained their freshness until day 11. In the uncoated control group, a 12.64% weight loss was observed by day 12, while a 5.22% weight loss was observed in the coated fruits [75]. In summary, AgNPs synthesized from cassava peel, when coated with gelatin, protected the fruits from UV radiation and exhibited antimicrobial activity. These effects significantly extended the shelf life of the fruits. Therefore, the use of AgNPs produced by green synthesis methods in combination with gelatin shows high potential for extending the shelf life of fruits.

### 3.2. Vegetable

In a particular study, CS–gelatin–AgNPs composite was developed as a biodegradable packaging material. AgNPs synthesized from *Mussaenda frondosa* leaves using green synthesis approach and DPPH tests showed that the antioxidant activity of AgNPs could reach up to 69% at maximum concentration of 200 μg/mL. In addition, the antibacterial effect of AgNP also measured and an increased antibacterial effect was observed with increasing amounts. TEM results used during characterization studies have shown that AgNPs are spherical and crystalline in shape and 10 to 30 nm in size. Also, different parameters such as TS, EAB, surface functionalities, and surface morphologies for all concentrations have been measured. Solution casting method has been used to prepare CH–Gelatin (GE)–AgNPs composite films with different concentrations of AgNPs, such as 0%, 0.0075%, 0.0125%, 0.025%, and 0.05%, referred to as CG, CG1, CG2, CG3, and CG4, respectively. Moreover, the biodegradability of the nanocomposite was measured, and after approximately 14 days, the composite showed the highest degradation rates. However, it should be highlighted that increased amount of AgNPs reduced the degradation rate with its antibacterial activity. To analyze the packaging capability, carrot pieces were coated with polyethylene (PE), CG (CH/GE), and CG4. The CFU/mL value of the carrot piece coated with CG4 was 10 × 10^10^, which was the best CFU/mL value obtained, followed by CG and PE, respectively [76]. The composite produced by the researchers confirmed that the film could be used in innovative and eco-friendly food packaging applications.

In another study, AgNPs were included in the composite containing microcrystalline cellulose (MCC), polyethylene glycol (PEG-1500), protein, and starch. AgNPs have been synthesized from *Azadirachta indica *leaves and characterization tests revealed that sizes of AgNPs synthesized are approximately 20 nm. Additionally, the NPs obtained after AgNP synthesis were subjected to antibacterial tests (disk dispersion) with both Gram-positive and Gram-negative bacteria, which can also be classified as food pathogens, and the highest antibacterial effect was against *E. coli*, while it did not show any effect against *Proteus vulgaris*. However, it was revealed that it was effective against most foodborne pathogens. To produce films, there are four different film matrices, such as [MCC and acetic acid] (referred to as (1)), [(1) + AgNPs] (referred as (2)), [(2) + (PEG-1500) + starch] (referred as (3)), and finally [(3) + whey protein], to measure different parameter changes according to ingredients of film but for the preservation test, MCC/starch/Whey/AgNP film has been used. To obtain how effective this film is as a food preservation material, researchers coated brinjal with the film. In a few days, brinjal samples that have no preservation application start to rot but brinjal with film shows longer shelf life compared to no-film samples. The composite film-coated brinjal did not lose a large amount of mass, which means that the shelf life required for the eggplant to rot has increased [77]. This composite film, which was produced as an effective way to prevent foodborne diseases caused by food spoilage, has become a creative alternative.

Saravanakumar et al. investigated how AgNP–polyvinylpyrrolidone-based glycerosomes affect the shelf life of the bell pepper *Capsicum annuum *L.* var. grossum* (L.) Sendt. Researchers utilized both chemical and green synthesis for the production of AgNPs. The films obtained by green synthesis were labeled G-PVP-AgNPs, while the films obtained by chemical synthesis were labeled C-PVP-AgNPs. It was observed that the average size of G-AgNPs was 37.01 nm, while after PVP and glycerol coating, G-PVP-AgNPs reached 123 nm. C-PVP-AgNPs had a size of 45.26 nm. MIC and minimum bactericidal concentration (MBC) tests were also applied to measure the antibacterial activities of the composites formed. *Salmonella enterica *(*S. enterica*), *P. aeruginosa*, *E. coli*, *B. cereus*, and *S. aureus *bacteria have been tested. The MIC for *S. enterica* was measured as 8.54 µg/mL with G-PVP-AgNPs and 11.25 µg/mL with C-PVP-AgNPs. Similarly, the MBC values were 55.61 µg/mL and 75.22 µg/mL, respectively. The lowest MIC value for G-PVP-AgNPs was found to be 5.45 µg/mL in *S. aureus*, indicating the highest antibacterial effect. For all other pathogens, G-PVP-AgNPs showed that it was a more effective antimicrobial agent by providing lower values for both MIC and MBC. As a result, the statement that G-PVP-AgNPs showed a stronger effect against all bacteria than C-PVP-AgNPs is a correct statement. To understand how G/C-PVP-AgNP films affect the shelf life of fresh-cut pepper, different parameters were measured for 15 days. First of all, similar levels of moisture content were observed among all groups, and the highest value was measured as 16.77% in the C-PVP-AgNPs group and the lowest as 16.42% in the control group. Total bacterial count (TBC) was the lowest in peppers coated with G-PVP-AgNPs with 419.22 CFU/g; this value was found to be 599.03 CFU/g in the C-PVP-AgNPs group and 928.08 CFU/g in the control group. In terms of Total Fungal Counts (TFC), the lowest load was seen in the G-PVP-AgNPs coating (553.59 CFU/g), and this value almost tripled in the control group and reached 1438.78 CFU/g. The highest hardness value was observed as 2650 g in the G-PVP-AgNPs-coated pepper and the lowest as 2252 g in the control group. The peppers coated with G-PVP-AgNPs maintained their elasticity for longer, indicating a fresher and more vibrant product. In the control group, the elasticity value was lower, reflecting textural loosening over time. When examining the fracturability values, the peppers treated with G-PVP-AgNPs maintained a high level of fracturability at 2659 g, indicating that the product maintained its freshness and structural integrity. In the control group, the fracturability value decreased to 2275 g, indicating structural weakening over time. To test how toxic the nanocomposite films were, pepper juices with and without nano-coating were given to *Caenorhabditis elegans* worms and survival rates and cellular damage were examined by fluorescent staining. In this test, pepper juices coated with G-PVP-AgNPs and C-PVP-AgNPs did not exhibit any toxic effects. However, the group receiving uncoated pepper juice exhibited severe cellular damage to the worms, and survival rates were significantly reduced. Furthermore, no toxic effects were observed in the positive control group receiving fresh pepper juice or in the group fed only *E. coli *[78]. To summarize the results, in this study, edible nano-coatings prepared with green, chemically synthesized AgNPs (G/C-PVP-AgNPs) were experimentally demonstrated to extend the shelf life of fresh-cut peppers, reduce microbial contamination, and exhibit no toxic effects. This produced film also has significant potential to protect human health.

In another study conducted by Singh et al., the potential of cellulosic packaging impregnated with AgNPs to extend the shelf life of vegetables and prevent microbial contamination was investigated. Initially, the dominant microorganism isolated from spoiled tomato and cabbage samples was characterized as *Aeromonas hydrophila *(*A. hydrophila*) through molecular analysis and 16S rRNA gene sequencing, showing 100% similarity to *A. hydrophila*. AgNPs were synthesized using *Camellia sinensis* using a green synthesis method, and reduction in AgNPs was confirmed by color change. The synthesized AgNPs had an approximate diameter of 35 nm, and they were characterized using UV-Vis spectroscopy, TEM, and Fourier Transform Infrared Spectroscopy (FTIR) analysis. An MIC test was performed to determine the antimicrobial activity of the AgNPs, and the results showed an MIC value of 15.3 mg/mL. A color-change-based viability test was also performed using Triphenyl Tetrazolium Chloride (TTC), and no color change was observed (indicating the absence of viable bacteria). Additionally, bacterial growth was completely inhibited when 100 µL of AgNPs were used, and a decrease in colonization was observed as the dose increased. To produce the film, a colloidal solution containing 10% AgNP was sprayed onto cellulosic paper. In the antimicrobial diffusion test applied to the film, squares cut from these papers were placed on *A. hydrophila* cultures on agar medium, and inhibitory zone formation was clearly observed. This result demonstrated that the produced packaging was able to inhibit bacterial growth. In the real-time experiments, tomato and cabbage samples were stored in AgNP and control group packages at room temperature for 7 days and were examined daily for appearance, odor, pigment loss, and microbial presence. While vegetables stored in AgNP-containing packaging retained their freshness, rotting and foul odors were observed in samples from the control group. Furthermore, AgNP-containing packaging largely prevented moisture loss; by the end of day 7, the moisture content of tomatoes was measured as 89.15% in the AgNP-containing group and 62.5% in the control group. The moisture content and nutritional profile of vegetables in packages containing AgNPs were preserved because no loss of cell permeability was observed in these samples; however, the main cause of cell damage is loss of permeability due to lipid peroxidation, which usually occurs under adverse environmental conditions [79]. In conclusion, this study demonstrated that AgNP films provide strong protection for different vegetables and significantly help preserve their nutritional value when applied at optimal ratios.

Another comprehensive study was conducted by Rasheed et al., where *Aspergillus niger *(*A. niger*) strains were used to synthesize AgNPs. Various characterization studies were conducted, and AgNPs were incorporated into the film. The films with and without AgNPs were then characterized for thickness, moisture content (MC), and water permeability (WP). The results showed that the addition of AgNPs reduced film thickness, reduced moisture content, and facilitated the production of films with lower water permeability. Subsequently, the deteriorated surfaces of decomposed liver, gizzard, apple, and orange samples were swabbed in culture media to isolate different bacterial and fungal strains. A total of three different bacteria (SR1, SR2, and SR3) and three different fungi (SF1, SF2, and SF3) were isolated. AgNPs exhibited highly effective antibacterial activity against both Gram-positive and Gram-negative bacteria, although they were observed to be more effective against Gram-positive bacteria. The highest inhibition against fungi was observed in *A. flavus* (15 mm), while the lowest inhibition was observed in *A. tamarii* (10 mm). In antibacterial tests of emulsions, emulsions containing AgNPs produced significantly higher inhibition zones against both bacteria and fungi than those without AgNPs. Against *S. aureus*, the inhibition zone was observed as 9 mm in the emulsion without AgNPs, while it was observed as 14 mm in the emulsion with AgNPs. In the films, AgNP-containing films also exhibited significant antibacterial activity, but were not as effective as the emulsions. Consequently, the highest antimicrobial activity was observed in the AgNP-containing whey emulsions. Finally, taro root and peas were coated with emulsions with and without AgNPs and with AgNP-containing films. The highest microbial load values were observed in the unwrapped control groups. In pea samples, whey protein emulsions containing AgNPs provided complete inhibition of all tested microorganisms, with a microbial load of 0 log CFU/mL measured in all four culture media. This clearly demonstrates the potent antimicrobial effect of AgNPs and demonstrates that the emulsion form establishes more effective contact with the surface, completely inhibiting microbial growth. In contrast, although film coatings with and without AgNPs significantly reduced microbial load, they failed to completely eliminate fungal and total bacterial loads and exhibited lower levels of protection than emulsions. In taro root samples, whey protein emulsions containing AgNPs provided complete inhibition in all four culture media, with microbial loads reduced to 0 log CFU/mL in all conditions. This result demonstrates that AgNPs are highly effective against both bacterial and fungal contaminants found on the taro root surface, and the emulsion form is highly effective in terms of surface coating. On the other hand, AgNP-containing films significantly reduced microbial load but still allowed for low microbial presence in some environments. Other samples examined in a similar manner included strawberry, green chili, carrot, and guava. In these samples, emulsions containing AgNPs were observed to exhibit the strongest antimicrobial activity against all tested microorganisms. In many environments, microbial loads decreased to 0 log CFU/mL, indicating complete inhibition. In contrast, emulsions without AgNPs, and especially AgNP-free films, reduced microbial load, but this success was more limited; in some environments, low levels of colony growth were still observed [80]. The findings revealed that AgNP-containing whey protein emulsions are highly effective in preventing microbial contamination on fruit and vegetable surfaces. Therefore, AgNP-containing emulsions offer a strong alternative for active and edible coating materials in terms of food safety and shelf life.

### 3.3. Meat

Another study reported that berry wax (BYW) usage in CT/AgNP composite is a notable alternative for biodegradable films. First, to synthesize AgNPs, petals of the *Paeoniaceae* plant were used, and results showed that AgNPs are 55 nm in size, and spherical in shape. To produce test groups, five different test groups that have different concentrations of AgNPs were prepared. These test groups contained AgNP at the rates of 0.5%, 0.75%, 1.0%, 1.25%, and 1.5%, respectively. According to thermal stability tests (TGA/DTG), the films became more heat resistant with AgNPs, and the highest degradation temperatures were seen at 1.0% and 1.25% AgNPs, approximately 415–416 °C. Mechanical properties of the film have been measured under different parameters. Significant increases have been observed in thickness, TS, and EAB with increasing AgNP concentration. The DPPH method was used to test the antioxidant activity of the films and it was found that the highest antioxidant activity belonged to the film with the highest amount of AgNPs (1.5%). Additionally, antibacterial activity of the films has been tested via *E. coli *and *S. aureus* as a Gram-negative and Gram-positive bacteria, respectively. Results revealed that film that contains 1.25% AgNPs has the most antibacterial activity for *E. coli* and for *S. aureus*, and the film containing 1.5% AgNPs showed the highest antibacterial activity. Moreover, SEM showed that the membranes of bacterial cells treated with composite films containing AgNPs were damaged. Finally, tests were conducted to extend the shelf life of rabbit meat at 4 °C and the efficiency of the composite film produced with different parameters was tested. When rabbit meat was packaged with AgNP-containing film, the brightness value and redness value were preserved longer; however, a lower rate of yellowing was observed. In the control group, the Total Volatile Basic Nitrogen (TVB-N) value exceeded the degradation limit by reaching 21 mg/100 g as of the 8th day. In the film group, this value remained below 19.25 mg/100 g even at the end of the 16th day. In the meat of the control group, the number of viable bacteria (TVC) was measured as 6.44 log CFU/g at the end of the 8th day. In the film group, this value was only 5.64 log CFU/g even on the 16th day. Finally, in TBARS tests, lipid oxidation in the control group reached 2.18 mg MDA/kg on day 8, exceeding the degradation limit. In the film group, this value remained at 1.91 mg MDA/kg on day 16, below the degradation limit [81]. To sum up, the research team has produced a composite film that is both biodegradable and highly effective, suitable for green synthesis methods, to extent the shelf life of foods, especially meat-type foods.

The study of Gasti et al. is an important study in the field of preservation of red meat products. They aimed to produce CS/PVA/AgNP composite films in a one-pot and environmentally friendly manner by reducing Ag^+^ ions using *Spondias pinnata* fruit pulp (SPFPE) and determine which of the composite films are the best for preservation of red meat. They designed four different test groups such as CS/PVA, CPPFE (CS/PVA + SPFPE), CPAg-1 (CS/PVA + SPFPE + 0.15 wt% AgNO_3_), and finally CPAg-2 (CS/PVA + SPFPE + 0.45 wt% AgNO_3_). Mechanical tests, such as TS, Young’s modulus (YM), and EAB, applied to the different composite samples and research, determined that CPAg-1 showed the highest TS and YM values; however, the TS value of CPAg-2 was decreased, YM was preserved, and EAB was decreased more compared to CPAg-1. Moreover, moisture absorption (MA), moisture retention capacity (MRC), water solubility (W_S_), and soil burial degradation (S_D_) (biodegradation) tests were applied and determined that CPAg-2 had the least moisture absorption and water solubility values and also had the highest moisture retention capacity. Also, CPAg-1 showed better S_D_ values than CPAg-2. This shows increasing concentration of AgNPs in composites causes worse S_D_ values. In addition, the importance of the WVTR test should not be overlooked in the production of food packaging materials, as researchers have also applied this test. As a result, they found that CPAg-2 showed the best barrier effect among other test groups with a value of 25.14 g/m^2^. The crystalline nature of CPAg-2 may be the reason for this high value. The overall migration limit (OML) test, which is conducted for reasons such as consumer health and protection of food quality, was also applied to the produced composites, and both the CPAg-1 film and the CPAg-2 film passed the tests. Before real-time tests, antimicrobial activity and antioxidant activity tests were applied. The most antioxidant effect is shown by CPAg-2 composite. In addition to this, CPAg-2 had the highest inhibition zone for all pathogenic bacteria, followed by CPAg-1. Finally, four different test groups were created with lamb meat and these groups were examined for 30 days. Initially, there were approximately 3.2 log CFU/g bacteria in all test groups. In the unwrapped sample, 7.6 log CFU/g bacteria were observed on the 6th day, which means that it was spoiled and could not be consumed. In CS/PVA- and CPPFE-wrapped meat, although microbial growth was slower, it was observed that it was spoiled on the 15th day. On the contrary, meat wrapped with CPAg-2 remained just below the spoilage limit with a value of 6.8 log CFU/g on the 30th day [82]. This shows that CPAg-2 composite film is not only an innovative alternative in extending the shelf life of red meat, but also can significantly prevent spoilage by reducing lipid oxidation to a considerable extent.

The work of Aouay et al. offers valuable insights into improving the shelf life of chicken meat products. To produce a composite to develop the shelf life of meats, first AgNPs were synthesized using cellulose nanocrystals (CNCs) but before that, CNCs were oxidized for 48 h and obtained ox-CNCs. In Zetasizer measurements, sizes of ox-CNCs-AgNPs determined as 245 nm and also surface charge was observed to be negative. It was analyzed by TEM that CNCs had a rod-like structure and AgNPs were bound to CNCs, but this binding was not homogeneous. Finally, for the characterization tests, XRD and TGA analysis have been applied and results of these analyses showed that there is a decreased moisture retention of CNCs-AgNPs. Also, increasement in the thermal stability of CNCs-AgNPs has been observed. It was also determined that there was an ash content of 20%. To test the antibacterial effect of CNCs-AgNPs, *S. aureus*, *Micrococcus luteus* (*M. luteus*), *Enterococcus faecalis* (*E. faecalis*), *Listeria monocytogenes*, *E. coli*, *P. aeruginosa*, *Klebsiella pneumoniae*, and *Salmonella enterica* bacteria were used as Gram-negative and positive bacteria. Different concentrations of CNCs-AgNPs such as 1%, 2%, and 4% (10, 20, and 40 mg/mL) were prepared. In the test, using the disk diffusion method, it was observed that the inhibition effect increased in a dose-dependent manner, while in broth medium tests, complete inhibition was observed in all bacteria at all doses of 3.2 mg/mL and above. In MIC tests, the lowest MIC value was observed in *M. luteus *and *E. faecalis*, while the highest MIC values were observed in *E. coli*, *K. pneumoniae*, and *L. monocytogenes*. It is important to predict that CNCs-AgNPs will also have antibacterial effects when used in biofilm or composite film form. To form PLLA/CNCs-AgNPs composite films, the solvent-free melt extrusion method was used and while one of the test groups have 2% CNCs-AgNPs, other test group have 4% CNCs-AgNPs and additionally, PEG was used as a dispersing agent and plasticizer. The same method was used for producing PVA/CNCs-AgNPs composite films and, as expected, there was one control group for each composite to apply different tests. Mechanical tests showed that CNCs-AgNPs affect the tensile strength positively but CNCs-AgNPs were better distributed in PVA and the durability of thin films has increased significantly. Although PLLA-PEG/CNCs-AgNPs films were also reinforced, they were not as effective as PVA due to dispersion limitations. In addition to the first antibacterial tests, composite films were also tested. Consequently, PVA-CNCs-AgNPs (4%) had the highest value of inhibition zone radius against all bacteria. PLLA-CNCs-AgNPs (4%) created smaller inhibition zones compared to PVA-CNCs-AgNPs (4%). Inductively, coupled plasma (ICP) analysis was also applied and silver release was below the limit established by European Food Safety Authority (EFSA). Finally, for the real-time tests, two chicken breasts wrapped with PVA-CNCs-AgNPs and neat PVA was studied and stored for 7 days at 4 °C and different parameters were measured. TVC at the 7th day was measured as 5.58 log CFU/g from PVA-CNCs-AgNPs film and it was below the spoilage limit. In contrast, chicken wrapped with neat PVA had 6.1 log CFU/g bacteria. There was also a significant decrease in the number of psychrophilic bacteria. PVA-CNCs-AgNPs film also suppressed the growth of intestinal contaminants. After 10 days of contact, the remaining Ag amount in chicken meat was found to be 0.035 mg/kg (according to ICP analysis), which is still below the value determined by EFSA [83]. As a result of these studies, the PVA-CNCs-AgNPs composite film extends the shelf life of chicken breasts and provides a highly innovative and alternative method for creating healthier storage methods for consumers and producers.

Li et al. produced pectin–gelatin–curcumin–AgNP (P-G–C–A) composite film and analyzed its preservative effect on shrimps. To prepare films, gelatin and pectin were mixed at a ratio of 1:1 and glycerol was added as the plasticizer agent. Curcumin solution and increasing proportions (0%, 0.05%, 0.5%, 2.5%, and 6.25%) of AgNO_3_ were added. After the resulting films were dried, they were abbreviated as P-G-C-A (added AgNP ratio). There were three types of films: P-G-C-A (0.05, 0.5, 2.5, and 6.25), P-G-A0.5, and P-G-C0.3. It was observed by TEM that the produced AgNPs were approximately 20 nm in size, and it was observed by SEM and energy-dispersive X-ray spectroscopy (EDS) that AgNPs were homogeneously distributed in films containing up to 0.05% AgNPs, while irregular structures were observed in films containing more than 0.5% AgNPs. In XPS analyses, it was observed that curcumin and gelatin played a role in the reduction in Ag^+^ ions. Different analyses were made for mechanical properties, the first of which was the test of color and light barrier properties. As a result of this analysis, it was observed that increasing the AgNP ratio turned the yellow-colored films into dark brown. Also, increasing AgNP enhanced the film opacity. In the WVP test, it was observed that the presence of AgNP and curcumin negatively affected the WVP value. The lowest WVP value was observed in the P-G-C-A0.5 film. On the contrary, the highest WVP value was observed in the P-G-C-A6.25 film. The highest TS and EAB values were measured in the P-G-C-A2.5 film, and TS and EAB decreased in the P-G-C-A6.25 film. In addition, WCA measurements were also conducted and it was understood that all surfaces of all films were hydrophobic, which is suitable for use in food packaging. To test antioxidant activity, DPPH and ABTS analysis was conducted and it turns out that P-G-C-A6.25 film demonstrated potent antioxidant activity, while P-G-A0.5 showed the lowest activity. Moreover, their antibacterial effects were tested on several bacteria strains and P-G-C-A0.5 showed 100% inhibition for *S. aureus* while P-G-C-A2.5 showed 100% inhibition for *E. coli*. One reason why this produced film is innovative is its ability to change color according to pH changes. When the pH is between 3 and 8, the film shows yellow, in the range of 9–10, light red, and in the range of 11–12, it shows dark red. It was also emphasized that this pH-dependent color change is a feature added by curcumin. P-G-C-A0.5 films were used as real-time experiments and these films were kept at room temperature for 5 days and examined to investigate shrimp spoilage. First, the color change in the film was highlighted. The initially yellow composite films were observed to have changed to a light orange color, which was attributed to the increase in pH caused by volatile nitrogen compounds. The pH value also increased from 6.7 to 8.6 during this period, which confirms the deterioration. It was observed that the produced film slightly extended the shelf life of shrimp at room temperature, but the emphasis was on monitoring spoilage by facilitating the monitoring of pH value, not on the shelf-life-extending effect of the film [84]. In conclusion, it should be highlighted that the produced P-G-C-A0.5 film is an innovative composite in the field of “smart packaging”, in addition to its antibacterial and antioxidant effects, thanks to its pH-responsive feature, which allows for monitoring degradation.

Wu et al. examined the incorporation of laurel essential oil (LEO) and AgNPs into a CS-based film using liposomes to achieve active preservation in food packaging applications. AgNPs and LEO were synthesized using lignin in a green synthesis manner and were encapsulated into nano-liposomes. The resulting Lip-LEO-AgNPs were added to a CS solution, and this mixture was coated onto a PE film to form PC-Lip/LEO/AgNPs. For comparison, PE-only and CS-coated PE (PE-CS) films were also prepared. TEM, SEM, and High Angle Annular Dark Field (HAADF) analyses were used in characterization studies. Additionally, EDS and zeta potential tests were performed, and these analyses revealed that the AgNPs were stable and had a 57% encapsulation rate. These analyses also revealed that the contents of the film were homogeneously distributed within the film matrix. The release of LEO and AgNP from the liposome was also tested, and these tests revealed a total release of 16.39% LEO in the first 8 h and 29.30% after 7 days. Only 11.79% of the AgNPs remained free after 7 days. These results indicate that liposomes are primarily responsible for the slow release of LEO and AgNPs. Antioxidant tests included DPPH, FRAP, and Total Radical-Trapping Antioxidant Parameter (TRAP). These tests revealed that the films were highly antioxidant thanks to LEO. Furthermore, the Lip-LEO-AgNPs film showed the highest antioxidant activity. The antibacterial activity of the developed films was tested on *S. aureus* and *E. coli*. In experiments using the agar diffusion method, PE and PE-CS films did not exhibit any antibacterial property, while the PC-Lip/LEO/AgNPs film created distinct inhibition zones against both bacteria. A higher activity was observed, particularly against *S. aureus*. Finally, it was evaluated whether the produced films could preserve the sensory, physical, and chemical properties of pork for 21 days at 4 °C. In the analyses, parameters, such as color, aroma, viscosity, elasticity, and broth quality, were examined, and while the control group meat became unusable after the 9th day, samples packaged with PE-CS film remained fresh for 12 days and samples packaged with PC-Lip/LEO/AgNPs film remained fresh for up to 15 days. pH measurements also provided findings supporting the quality; while the spoilage limit of 6.7 was exceeded with 7.11 in the control group, the PE (6.79) and PE-CS (6.67) groups also exceeded the limit over time. On the other hand, the pH value in the PC-Lip/LEO/AgNPs group remained at 6.54 and remained below the spoilage threshold. The lowest TVB-N values, which are considered an indicator of chemical spoilage, were observed in the PC-Lip/LEO/AgNPs group; while the control group exceeded the legal limit of 15 mg/100 g on day 6, the PE group on day 9, and the PE-CS group on day 12, the PC-Lip/LEO/AgNPs group exceeded this limit only on day 15 [85]. At the end of the studies, it was determined that PC-Lip/LEO/AgNPs film is a promising alternative food coating material for red meat foods and their derivatives.

Gellan gum (GG) was used as both a reducing and stabilizing agent during the synthesis of AgNPs in a study by Xhai et al. The formation of AgNPs was confirmed by a color change resulting from the mixture of AgNO_3_ and GG. Subsequently, in a test conducted to monitor the release of H_2_S gas, which occurs when meat spoils, using smart packaging technologies, the GG-AgNP turned from yellow to colorless upon encountering H_2_S, a color change confirmed by the instrument. Consequently, the system sensitively and reliably detected even very low amounts of H_2_S. Furthermore, stability tests observed the stability of the GG-AgNP system over time and in salty environments. The system remained stable for 30 days and maintained its stability up to 0.1 M NaCl, demonstrating its appropriate shelf life and durability. Studies to optimize pH tested the stability of the GG-AgNP solution at different pH levels and how its H_2_S detection capacity varied with pH. Analysis showed that the sensor was unstable below pH 5 but stable above it. The best H_2_S detection was at pH 7, so pH = 7 was chosen as the optimal pH for the GG-AgNP solution. The selectivity of GG-AgNP to H_2_S was subsequently tested, and it was observed that none of the volatile substances that can form during meat spoilage (except methanethiol) caused a significant color change in the GG-AgNP solution. Only methanethiol, due to its weak interaction with silver, showed a slight decrease, but even this was very low compared to H_2_S. In a test conducted to evaluate the H_2_S-sensing capacity of the hydrogel form of GG-AgNP and its suitability as a portable sensor, a portable gelled system was prepared by adding agar to the GG-AgNP solution. This hydrogel lost its color noticeably within approximately 5 h of exposure to H_2_S vapor. The color change within the gel was measured numerically using the B value, which represents a blue hue. Consequently, a strong linear relationship was established between the H_2_S concentration and the B value. Furthermore, when the hydrogel was tested with various volatile compounds, it did not change color, confirming its selective response only to H_2_S. Finally, silver carp were used to test whether the produced hydrogel could truly indicate degradation. The hydrogel was stored at 4 °C with fish samples, and color change began on day 4, and the presence of H_2_S was visually confirmed on day 8. Digital analyses showed a steady increase in the B value, and TEM imaging revealed the Ag@Ag_2_S structure. A strong correlation was established between the TVC and the number of H_2_S-producing bacteria and the B value, demonstrating that the sensor can sensitively and reliably monitor degradation, particularly in relation to H_2_S production. Additionally, similar results were obtained with chicken breast samples as with silver carp [86]. In conclusion, this study demonstrates the potential of a GG-AgNP-based color-change-sensitive system for real-time, selective, and device-free monitoring of meat spoilage, making a significant contribution to smart food packaging applications. Considering the portability, low cost, and ease of production of the hydrogel form, GG-AgNP hydrogels have strong commercial viability. Its ability to specifically detect H_2_S and its reliable performance in various meat types differentiate GG-AgNP hydrogel from conventional methods and demonstrate its potential as a new solution in the field.

Cao et al. investigated the synergistic effects of potato-oxidized hydroxypropyl starch (POHS), pectin (P), Clitoria ternatea anthocyanin (BA), and AgNPs to present an innovative approach to biodegradable and smart packaging systems. Initially, POHS and pectin were mixed to form a polymer matrix for film formation, and this matrix was then completed by adding BA and AgNPs at different concentrations. SEM, TEM, and AFM tests revealed homogeneous distribution of the NPs on the matrix surface. UV-Vis tests indicated that the addition of BA conferred UV-protection properties on the film. Mechanical tests revealed that TS increased with the addition of AgNPs, and BA increased flexibility. Furthermore, the addition of AgNPs increased WVP, while the addition of BA at the optimum level positively affected WC. Subsequently, *E. coli* and *S. aureus* bacteria were used for antimicrobial tests, and the POHS/P/BA/AgNPs film demonstrated strong inhibition zones against these bacteria. DPPH tests were also performed to measure antioxidant activity, and these tests demonstrated radical scavenging activity of the nanocomposite. Additionally, to test its suitability for smart packaging, the colors of the BA were observed at different pHs. At pH 2, it turned pinkish, while at pH 11, it turned greenish. Furthermore, to serve the same purpose, the color change of the POHS/P/BA/AgNPs film against volatile ammonia was observed, with purple in the first minute and varying shades of green at 18 min. Finally, this nanocomposite film was tested on beef using various parameters (such as TVB-N, TVC, TBARS, and pH). The results of the tests revealed that the meat exceeded the freshness limit on day 7. The color change showed high correlation with the actual chemical spoilage parameters of the meat [87]. This study tested the comprehensive protective properties of the new POHS/P/BA/AgNPs film, which provides smart packaging and shelf-life extension, and demonstrated its protective properties.

### 3.4. Others

#### 3.4.1. Dairy

Kumari et al. produced Ag/Carr (silver/carrageenan) nanocomposite films and applied them to cheese and strawberries and investigated how Ag/Carr affected the shelf life and its protection. The produced films were named Ag/Carr-30, Ag/Carr-60, and Ag/Carr-90, corresponding to their concentrations of 30, 60, and 90 mL, respectively. The mechanical properties of the produced film were tested and, as a result, it was observed that the thicknesses varied between 40.7 μm and 43.5 μm in direct proportion to the concentration. While the TS value was 30.01 MPa in Ag/Carr-30, this value has been measured as 39.83 MPa in Ag/Carr-90 film. The Elastic Modulus (EM) value was measured as 2.60 in Ag/Carr-30 and 3.06 GPa in Ag/Carr-90 film. In thermogravimetric analysis, it was observed that the degradation of carrageenan and glycerol occurred at approximately 300 °C, and the thermal stability increased significantly with the increase in AgNPs. In the oxygen permeability (OP) tests, it was concluded that Ag/Carr-90 film was the most effective film in preventing food oxidation. In the MC measurements, it was observed that the increased AgNP amount reduced the MC, which indicates a better shelf life. In the total soluble matter (TSM) tests, it was also observed that the increased AgNP amount enhanced the hydrophobicity feature. Finally, in the WVP analyses, it was concluded that NPs reduced water permeation and increased preservation. In addition, the biodegradability levels of the samples were measured and it was observed that they were completely degraded in the soil in 4 weeks. In the antimicrobial activity tests, the disk diffusion method was conducted and *E. coli* and *S. aureus* were preferred as bacteria (Gram-negative and -positive). As a result, although all films had significant antibacterial effects, Ag/Carr-90 was the film with the biggest inhibition zone among them. Finally, cottage cheese and strawberries were used for real-time tests. In the cheese test, cheeses in containers covered with Ag/Garr film were examined for 10 days. Following 10 days, MC remained almost constant and no change was observed in pH value. Structural integrity was also preserved after 10 days. While color, taste and smell were as they should be until the 9th day, signs of deterioration were observed on the 10th day. Cheese, which has an average shelf life of 3 days in plastic packages, showed completely stable properties until the 9th day when stored with Ag/Carr. Similarly, the packaged strawberries were stored at room temperature for 8 days and examined. Four parameters were measured as follows: total soluble solid (TSS), vitamin C, titratable acidity, and weight loss. The TSS value remained unchanged until day 3, then decreased from 20.9% to 20.6% by day 8. Vitamin C dropped by 8 g/kg, titratable acidity decreased from 0.2% to 0.18%, and a 0.5% weight loss was observed by day 8 [88]. As a result of the strawberry experiment, Ag/Carr films were able to extend the shelf life of strawberries, which had an average shelf life of 2 days, to 8 days, significantly delaying microbial growth and preserving their nutritional content.

Pouyamanesh et al. produced an innovative composite film and studied it on pasteurized and traditional butters. AgNPs at concentrations ranging from 0% to 17.5% were dispersed in polyethylene glycol monostearate (PGE) and incorporated into low-density polyethylene (LDPE) by the high-temperature melt blending method. The results showed AgNPs exhibited a particle size of 20.63 nm on average. It was also observed that the NPs were homogeneously distributed within the film. According to antibacterial analysis results obtained after 30 days of 4 °C storage, the TBC was measured as 229.66 × 10^4^ CFU/g in the pasteurized butter control group, while this value was completely eliminated (ND—Not Detected) in the 17.5% AgNP/LDPE film-coated group. In traditional butter samples coated with the same film, the TBC value decreased from 290.66 × 10^4^ CFU/g to 16.00 × 10^4^ CFU/g. For *S. aureus*, the load in the control group in pasteurized butter was 20.66 × 10^4^ CFU/g, while the 17.5% AgNP/LDPE film application completely eliminated this pathogen. In traditional butter, although the *S. aureus* load decreased from 270.66 × 10^4^ CFU/g to 54.00 × 10^4^ CFU/g, complete elimination was not achieved. While *E. coli* contamination in the control group showed 33.00 × 10^4^ CFU/g in pasteurized butter, the application of 17.5% AgNP/LDPE film reported complete elimination (ND) of this bacteria. In traditional butter, the* E. coli* load was reduced from 38.33 × 10^4^ CFU/g to 1.33 × 10^4^ CFU/g. Finally, psychrophilic bacteria were completely eliminated only in pasteurized butter (ND). The load in the control group was 171.66 × 10^4^ CFU/g. However, in traditional butter, the psychrophilic bacterial load was reduced from 290.66 × 10^4^ CFU/g to only 79.00 × 10^4^ CFU/g [89]. These data indicate that the application of 17.5% AgNP/LDPE film has the potential to completely eliminate microbial contamination, especially in pasteurized butter, but is not fully effective in conventional butter due to the high initial microbial load. In addition to microbial tests, different parameters related to mechanical properties were also tested. No significant change was found in moisture content in both pasteurized and traditional butters after 30 days, and film application was found to prevent moisture loss. In the context of solid non-fat (SNF) ratios, stable values were obtained in all groups and it was found that film coatings did not affect these components. It was found that the fat content remained constant between 82 and 84% in both butters and film application did not cause any change in the fat content. In acidity values (as a percentage of oleic acid), stable values were maintained in all groups and it was found that lipid hydrolysis did not occur. Finally, a slight decrease in saponification numbers (from 236.61 to 228.26 mg KOH/g) was detected in pasteurized butter with film application, indicating a change in the fat chain structure. In conclusion, while covering pasteurized butter with 17.5% Ag/LDPE film allows safe storage in the refrigerator for at least one month, the efficiency of this application is relatively lower for conventional butter due to its limited preservative effectiveness [89].

Braun et al. also tested the preservative effect of AgNPs on dairy products. However, these tests did not utilize full packaging methods; instead, they investigated the potential of AgNPs in milk packaging. They determined this preservative effect by measuring microbial activity and investigating the chemical interactions between milk and silver. They used three different methods to measure the preservative effect of AgNPs: mixing 15 nm AgNPs directly into milk, mixing silver phosphate glass directly into milk, and finally, adding AgNO_3_ to the sterile milk. pH monitoring was also performed to measure the antibacterial effect. First, to measure the antibacterial effect of silver, *Streptococcus thermophilus *was mixed with milk and inoculated. AgNPs at different concentrations (10, 50, 100, and 200 mg/L) and silver phosphate glass powders with silver contents of 8 mg/L and 32 mg/L were added to the inoculated milk. Results showed that AgNPs at concentrations of 200 mg/L and 100 mg/L significantly reduced milk acidification by inhibiting the growth of bacteria. Additionally, the effectiveness of AgNPs was measured at different temperatures and it was observed that AgNP at a concentration of 100 mg/L was effective at all temperatures, but temperature alone could not prevent bacterial growth. Results from measurements of the effect of silver phosphate glass, which slowly releases Ag into the medium, showed that bacterial growth was significantly slowed even at 8 mg/L total Ag. More importantly, the milk did not become acidic at 32 mg/L (no pH drop was observed over 7 days). In the potentiometric measurements of the tests where AgNO_3_ was added directly into milk, it was observed that at least 5 mg/L Ag^+^ should be present in the milk medium for effective antibacterial effect [90]. The results of these studies suggest that, while AgNPs may be able to prevent milk acidification and spoilage, their toxic nature limits their use. This experiment demonstrates that the practical use of AgNPs in shelf-life extension applications for milk and its derivatives is risky, but it also highlights their potential for incorporation into packaging technologies.

#### 3.4.2. Bakery

Another research about integrating AgNPs into composites in food packaging applications comes from Yazdi et al. In this study, bacterial cellulose nanofiber (BCNF), CS, and finally AgNPs at different concentrations were used to create the composite CS/BCNF/AgNPs. AgNPs (0.5%, 1%, and 2%) were dispersed into water by ultrasonic treatments and then mixed with CS solution and BCNF to produce the film. The resulting films were named CS/BCNF/0.5%AgNPs, CS/BCNF/1%AgNPs, and CS/BCNF/2%AgNPs. Characterization tests started with WVP and, as a result, it was observed that with the addition of BCNF and AgNP to the CS film, the value decreased from 3.75 × 10^−10^ g/smPa in the neat CS film to 0.85 × 10^−10^ g/smPa. Light transmission and opacity analyses showed that while the UV light transmission rate at 280 nm was 8.58% in the CS film, it decreased to 2.59% in the CS/BCNF/2% AgNPs film. Differential scanning calorimetry (DSC) analyses showed that thermal stability increased with the addition of AgNP and BCNF. In TGA analysis, three stages of degradation were observed as follows: 100 °C test and acetic acid evaporation, decomposition of CS and BCNF side chains at 250–300 °C, and finally degradation of carbon residue at 420–600 °C, but the addition of AgNP increased the minimum temperature required for degradation (CS: 206.13 °C, CS/BCNF/2%AgNPs: 227.44 °C). As the last characterization study, SEM analysis was applied and the results showed that the fibers and NPs were homogeneously distributed in the CS/BCNF/1%AgNPs film, but the CS/BCNF/2%AgNPs was rougher and the particles were agglomerated. For real-time experiments, bread samples were firstly coated with CS/BCNF/2%AgNPs solution with a sterile brush and packed in polyethylene bags and stored at room temperature for 15 days. The water activity (a_w_) and MC values of breads in each group were measured for 15 days. The fastest water loss was seen in the control group (uncoated bread). The best moisture content and lower a_w_ value were observed in the CS/BCNF/2% AgNPs coating. Crumb hardness was measured with a tester instrument between 0 and 15 days. Hardness increased over time in all samples. However, the lowest hardness value was observed in breads coated with 2% AgNP. Moisture-retaining coating slowed down staling. Finally, mold and yeast growth was measured. Samples taken from bread slices on days 1, 5, 10, and 15 were inoculated into potato dextrose agar medium. Mold–yeast growth was measured as 7.91 log CFU/g in the control group on day 15. No fungal colonies were observed (0 log CFU/g) in coatings containing 1% and 2% AgNPs for 15 days. These results confirmed the strong antifungal effect of AgNPs. In the second stage of the real-time experiments, CS/BCNF/AgNPs films poured into the petri dish and dried were wrapped around the bread samples and the wrapped breads were placed in polyethylene bags. These samples were observed at 25 °C for 60 days. Two different experimental sets were created, the first of which was artificially inoculated with *A. niger* suspension breads and the other was natural, non-inoculated breads. In the results of the first experimental set, mold started on the 4th day in the control group, while no mold was observed in the film-covered breads until the 28th day. In the second set of experiments, mold growth was observed on the 5th day in the control group, while no fungal growth was observed in the samples coated with CS/BCNF/2%AgNPs until the 38th day [69]. To sum up, this film stands out as an effective active packaging alternative for bread and similar products with its properties such as low water vapor permeability, high light barrier, improved thermal stability, and strong antifungal activity.

Gopalakrishnan et al. contributed to an important study on extending the shelf life of bread. The researchers used green-synthesized AgNPs using extracts from the peels of pomegranate and kinnow fruits. It was determined that the smallest AgNP formation occurred in the mixture containing 2 mM AgNO_3_, and this solution was used to form the films. Additionally, it was observed that AgNPs synthesized with pomegranate extract were both more crystalline and thermally stable. In Total Phenolic Content (TPC) analyses, the TPC values for both pomegranate- and kinnow-derived AgNPs were significantly higher than those from extracts alone. In Total Flavonoid Content (TFC) analyses, the flavonoid content in AgNPs was found to be significantly higher than in extracts. Furthermore, pomegranate AgNPs were found to contain higher flavonoid content than kinnow AgNPs. The DPPH method was used for antioxidant tests, and the results showed that AgNPs had significant antioxidant activity than the extracts. In addition, ABTS tests demonstrated that AgNPs (from both pomegranate and kinnow) exhibited significantly stronger radical scavenging activity than extracts. These AgNPs, along with pomegranate and kinnow extracts, were separately applied to cellulose-based papers, and the resulting films were designated PM-C (control group), PM-K (kinnow peel), and PM-P (pomegranate peel). The resulting films were characterized by thickness, color, gas and water vapor permeability, and water behavior. While the thickness and weight of all films were measured as the same, Oxygen Permeability Coefficient (OPC) and WVPC (Water Vapor Permeability Coefficient) values decreased significantly in PM-P and PM-K compared to PM-C, indicating increased gas and moisture impermeability. Water content (%), swelling degree (%), and solubility (%) values were also measured. All three parameters were found to be significantly lower in PM-P and PM-K compared to PM-C. Finally, SEM tests were conducted to observe the surface morphology of the films. This analysis revealed that PM-P contained smaller NPs, while PM-K contained larger AgNPs. Finally, the extent to which PM-C, PM-K, and PM-P films could delay microbial spoilage in bread was tested. Each piece of bread was packaged in a sterile environment, with the AgNP-coated surfaces in contact with the environment. Samples were stored at room temperature for 7 days and examined. No microbial growth was observed in all of the tested groups until day 5, while at day 7, the total bacterial load in the control group had increased to 40 CFU/g and the yeast/mold load to 10 CFU/g. In the PM-P and PM-K groups, both PC and YMC remained at <10 CFU/g. These results demonstrate that AgNPs form an effective antimicrobial barrier on the packaging surface [91]. In conclusion, cellulose packages modified with AgNP-containing PM-P and PM-K extended the shelf life by delaying microbial growth on the 7th day, thus showing potential as an effective antimicrobial solution in the packaging of perishable foods such as bread.

In conclusion, the potential offered by AgNP and AgNP-integrated preservative materials in extending the shelf life of food products and preventing microbial spoilage has been clearly demonstrated by studies conducted in various categories such as fruit, vegetables, meat, dairy, and bakery products (Table 1). AgNPs integrated into both active and smart packaging systems slow down quality losses by preserving the physical and chemical properties of foods, increasing consumer safety and shelf life. In sensor-based systems, AgNP addition offers an innovative solution for real-time monitoring and early warning for monitoring spoilage indicators such as color change and gas release. However, issues such as the toxic effects of AgNPs remain a matter of debate. Therefore, comprehensive approaches that also consider safety and biodegradability are needed in future studies.

## 4. Toxicity Concerns and Biocompatibility of AgNPs-Based Nanocomposites

AgNPs are used in food packaging applications because they can improve the protective features and mechanical and antimicrobial properties of films and also preserve the quality of foods [12]. In contrast, the potential harms of AgNPs to human health and the environment are still a matter of debate. It is known that the mechanisms mentioned in previous sections that confer antimicrobial and antioxidant properties to AgNPs can generally cause dose-dependent toxic effects. Studies have shown that AgNPs cause oxidative stress by increasing ROS levels, which in turn triggers both autophagy and apoptosis through autophagosome formation and activation of cell death pathways [125]. Furthermore, studies have also proven that the mechanisms of AgNP toxicity are due to higher intracellular Ag^+^ production leading to cytotoxic and genotoxic activities [126]. Moreover, it is a known fact that AgNPs can also cause lipid peroxidation, biomass reduction, photosynthetic efficiency reduction, phytoplankton assemblage, ribosome function, and disruption of energy production, highlighting the toxic properties of AgNPs [127]. For that reason, cytotoxicity tests, such as measuring Ag migration, DNA damage tests, MTT assays, etc., are required in producing food packaging materials.

Unlike physical and chemical synthesis approaches that often involve toxic reducing agents and high energy requirements, natural sources are utilized in green synthesis such as plant extracts, microorganisms, or biopolymers as both stabilizing and reducing agents [128]. This approach not only eliminates the use of hazardous chemicals, but also aligns with key green chemistry principles by prioritizing the prevention of waste at its source, encouraging the use of feedstocks derived from renewable biological resources, and promoting chemical synthesis methods that produce substances with minimal toxicity to human health and the environment. In the context of food packaging, the advantages of such green synthesis methods include the incorporation of biocompatible and environmentally friendly nanoparticles into packaging matrices, reduced risk of chemical residues migrating into food products, and compatibility with regulatory frameworks that increasingly favor sustainable and non-toxic production processes. Moreover, green-synthesized nanomaterials can exhibit improved functional properties such as enhanced antimicrobial and antioxidant activity, which are particularly desirable in extending shelf life and ensuring food safety. However, despite these advantages, several limitations should also be considered. Green synthesis processes can suffer from challenges in reproducibility, scale-up, and standardization due to variations in biological sources and synthesis conditions [129]. Additionally, while the use of natural extracts and microorganisms is appealing, controlling particle size distribution and achieving consistent physicochemical properties remain significant hurdles [130]. These limitations may restrict the translation of laboratory-scale findings into industrial-scale food packaging applications unless further optimization and standardized protocols are established. Therefore, while green synthesis represents a promising and sustainable alternative to conventional methods, ongoing research is needed to balance its ecological benefits with the technological and regulatory demands of the food packaging industry [131]. Furthermore, methods like these are typically performed under mild reaction conditions, such as using atmospheric pressure and ambient temperature, which contributes to energy efficiency by reducing the need for excessive heating or cooling applications, and supports safer chemical processes by minimizing the risks associated with extreme conditions. The biodegradability and biocompatibility of plant-based synthesis also facilitate safer integration into biodegradable packaging materials. Thus, green-synthesized AgNPs improves both the functionality and sustainability of active food packaging applications, contributing to extended shelf life and reduced dependence on synthetic preservatives [15,132,133].

Many studies have shown that the migration of silver from food packaging materials in solution or film form or containers into food may be a concern for the environment and public health [134]. Therefore, toxicity tests are of great importance in research studying the use of AgNPs in food contact materials. Green-synthesized AgNPs offer a great alternative for food packaging development. There are various studies focusing on the characterization of green-synthesized AgNP-based food packages.

In one study, xanthan/agar and nanocellulose-based biocomposite packaging using green-synthesized AgNPs with chamomile extract showed effective antimicrobial activity against *E. coli* and *S. aureus* [135]. In another study, green-synthesized AgNPs integrated into corn starch-coated paper packaging showed strong antimicrobial activity against *E. coli* and significantly extended the shelf life of cherry tomatoes and grapes [115]. A further study, provided by Amin et al., aimed to produce a composite film using AgNPs synthesized from *Ulva lactuca* extract. Subsequently, the synthesized AgNPs were used in conjunction with Ulvan to prepare a nanocomposite film. This non-toxic and green-synthesized nanocomposite successfully passed both antibacterial and antioxidant tests despite its low AgNP concentration [136]. At this point, the importance of green synthesis methods emerges because the AgNPs obtained by these methods have the advantages of being non-toxic, cheap, and environmentally friendly [137].

In one of the studies, antibacterial effects of chemically (Chem-Ag) and green-synthesized AgNPs (g-AgNPs) on *E. coli* were compared. Chem-Ag NPs suppressed bacterial growth more strongly in the early stages, while g-Ag NPs caused higher viability loss in the long term. This long-term effect was attributed to the slow Ag^+^ release of g-AgNPs or the natural antibacterial compounds on their surfaces. Consequently, g-AgNPs were found to be more suitable for long-term antibacterial applications [138]. Another study also evaluated that AgNPs synthesized from the extract of the *Osbeckia leschenaultiana* plant had moderate toxicity in an *Artemia salina* lethality assay [139].

Some studies have shown that size, shape, and surface charge can also affect toxicity. For instance, a study explaining the relationship between surface charge and toxicity found that increasing negative surface charge reduced toxicity, whereas positively charged NPs exhibited greater bactericidal activity than those of negatively charged NPs [140]. In another study conducted in this direction, zebrafish were found to be more sensitive to 20 nm AgNPs than to NPs with a size of 100 nm. This suggests that particle size may be a more dominant factor in toxicity than other physicochemical properties such as surface coverage alone. In particular, the larger surface area of smaller NPs may interact more strongly with biological systems, leading to more pronounced toxic effects at the cellular level [141]. In the model plant species *Lolium multiflorum*, nanocube AgNPs were observed to reduce root growth by only 5.3%, whereas spherical NPs were 39.6% more damaging. Consequently, nanocubes were observed to maintain antibacterial activity against bacteria, such as *E. coli*, *B. cereus*, and *P. aeruginosa*, while reducing environmental toxicity. Toxicity comparisons were also conducted in model organisms such as zebrafish and *Caenorhabditis elegans*. While nanocube AgNPs caused lower mortality and developmental disorders, spherical NPs exhibited higher toxicity. Significant impairments were observed in physiological parameters, particularly heart rate, body length, and swimming behavior in zebrafish. In tests on *Caenorhabditis elegans*, AgNPs in nanocube form had less negative effects on the organism’s growth rate and reproductive capacity. In contrast, spherical NPs increased oxidative stress levels and caused changes in gene expression [142].

Finally, biodegradability is another essential property of nanocomposites for food packaging products. Biodegradable materials can be defined as materials that function for a specific period of time during their lifespan and then degrade through various processes under regulated environmental conditions, causing minimal or no harm to the environment [143]. Biodegradability and biocompatibility tests are essential for various coating methods, particularly when producing nanocomposite solid coating materials.

To illustrate, Nguyen et al. left the C-AgNP film they synthesized from coffee in the soil for 120 days and observed that the film lost 85% of its mass, which is the biggest indicator of an eco-friendly film [144]. In another study conducted by Mathew et al., it was observed that films without AgNPs (PVA, PG (PVA + Guar gum), and PGM (PVA + Guar gum + Myrrh)) started to degrade on the 15th day and were completely degraded on the 60th day, while neat PVA and PAGM (contains AgNPs) films started to degrade on the 30th day and the degradation continued until the 110th day [145]. These studies show that although AgNPs significantly reduce degradation, AgNP-containing films can still be designed to be biodegradable.

Various methods are presented in the literature to reduce the toxic risks of AgNPs. The main objectives are to reduce their harmful effects on human health and the environment while simultaneously expanding their safe and sustainable applications for consumers and ecological systems. Among the proposed strategies, the use of green synthesis methods is noteworthy. While AgNPs obtained by chemical or physical synthesis methods can produce high amounts of toxic by-products, green synthesis using plant extracts, microorganisms, or biological materials offers safer alternatives with low energy consumption and an environmentally friendly approach [146]. Furthermore, it is possible to increase biocompatibility and reduce genotoxic effects such as DNA damage through surface modification of AgNPs. Indeed, PVP-coated AgNPs have been reported to exhibit lower toxicity than bare NPs [146]. Furthermore, since toxicity is largely dependent on particle size and dose, it is important to determine appropriate dose ranges and develop safety standards that reduce the risks of long-term low-dose exposure [147]. The green toxicology approach recommends adopting environmentally friendly methods not only in the production phase but also in the use and disposal processes to minimize toxic effects [147]. These strategies improve the safety of AgNP-based films for food preservation. Moreover, reducing the toxicity risks of AgNPs expands the applicability of these nanomaterials to food products for shelf-life extension or smart packaging studies. Consequently, the advantages of AgNPs or AgNP-based nanocomposites in the food packaging field can be maintained while minimizing potential adverse effects on human health and the ecosystem [12]. Thus, the advantages of AgNPs in food packaging can be maintained while limiting potential adverse effects on human health and the ecosystem.

To summarize, while AgNPs offer advantages, such as antimicrobial activity, extended shelf life, and physical durability, in food packaging, their toxicological effects remain a concern. However, these effects can be mitigated by particle size, shape, surface charge, and different synthesis methods. AgNPs produced through green synthesis are much less toxic and have a much higher biodegradability rate. Therefore, risk management actions, such as using low doses, ensuring biodegradability, controlling food contact, and environmentally friendly synthesis methods, are crucial to minimize the toxic effects of AgNP-containing nanocomposites.

## 5. Conclusions and Future Perspective

AgNP offers strong antibacterial and antioxidant properties, making them promising agents in active and smart food packaging applications. Various recent food preservation studies have shown that films or matrices incorporating AgNPs clearly reveal their high potential in protecting food from spoilage and contamination. In this review, food preservation and packaging studies carried out in recent years are discussed and the potential of AgNPs in food preservation applications and the reasons for this potential are explored.

Although it has been observed that AgNPs have positive effects on the nanocomposites in which they are incorporated and can create shelf-life-extending and biosensor effects, the dose-dependent toxic effects of AgNPs continue to be a matter of great debate. Consequently, in studies where AgNPs are included in substances that come into contact with food, information should be provided by testing by different analysis methods to determine whether AgNPs migrate into the food or reduce the biodegradable effect. In the future studies on food preservation containing NPs, it is very important to increase the in vivo and in vitro experiments and to test the release of silver into the food. Moreover, for more comprehensive and effective food preservation applications in the future, films in which AgNPs are integrated into different food types need to be tested under real conditions, such as for extending shelf life or validating sensor properties; such real-world trials are quite limited, especially in the field of smart packaging, and it is of great importance to increase them. Furthermore, while recent research have extensively tested fruit and vegetable-based foods; studies on other food types, such as dairy and bakery products, have been insufficient. At the same time, the need to extend the shelf life of beverage products and to develop smart packaging approaches remains an important consideration, consistent with current research trends in food preservation. In this context, in addition to the food types mentioned in the study, food types such as fruit juices, milk and dairy products, soft drinks, and fermented beverages constitute priority target groups due to their high sensitivity to microbial spoilage, oxidative processes, and physicochemical changes. Therefore, more intensive research into smart packaging strategies for these products, including nanotechnological solutions, and real-time quality monitoring systems, is critical for both food safety and industrial applications. Future studies focusing on these types of foods are crucial to broaden the applicability of these nanocomposite systems. In addition, it is of great importance to synthesize AgNPs using green synthesis methods in order to ensure that the packaging and protection systems produced are eco-friendly. Finally, consumer distrust that may arise due to the toxic effects of AgNPs can be supported by awareness campaigns. In this context, the use of AgNPs in food contact materials should be evaluated within the scope of strict legal regulations valid at the international level. Article 3 of Regulation (EC) No 1935/2004 states that such materials must not migrate any substances that could endanger human health, while Article 5 of Regulation (EC) No 2023/2006 requires compliance with good manufacturing practices (GMP). Furthermore, Article 6 and Annex I of Commission Regulation (EU) No 10/2011 stipulate that substances in nanoform may only be used where expressly authorized and listed under specific conditions. In addition, EFSA’s 2021 risk assessment guide for nanomaterials requires AgNPs to undergo detailed characterization, migration tests, and toxicological assessments before they can be placed on the market.

In conclusion, AgNPs, thanks to their effective antimicrobial and antioxidant properties, hold significant potential in the development of next-generation food packaging materials. However, utilizing this potential in a safe and sustainable manner is crucial for both human health and environmental impacts. Therefore, AgNP applications need to be evaluated not only in terms of applicability performance but also in terms of health and regulatory requirements.

## Figures and Tables

**Table 1:** Applications of AgNP-based packaging materials for different food products.

Food Type	Packaging Form	AgNP Integration Method	Physicochemical Properties of AgNPs/Material	Observed Effects	Reference
Cherry tomato	Pectin/AgNPs biofilm	AgNPs synthesized with NaBH_4_, blended into pectin solution and cast.	AgNP: 7–10 nm. Low methoxy pectin; strong gel network; AgNPs enhanced antibacterial, mechanical, and WVTR properties.	Shelf life extended to 10 days; preserved weight and sensory quality; antibacterial against various bacteria.	[92]
Tomato, coriander leaf	Blotting paper coated with green-synthesized AgNPs	AgNO_3_ + plant extracts on blotting paper; in situ green synthesis.	AgNP: 27–31 nm. Biodegradable paper; porous matrix; SEM/TEM verified AgNPs presence; antimicrobial coating.	Shelf life extended to ~30 days for tomato and ~15 days for coriander; effective antibacterial action against various bacteria strains.	[93]
Strawberry	CH + EOs (essential oils) + AgNPs films ± γ-irradiation	AgNPs, blended with CH-EO matrix via solution casting.	Smooth surface; improved tensile strength; stable WVP; FTIR showed matrix interaction.	Reduced weight loss and decay; improved firmness and phenolic content after 12 days storage; strong antimicrobial activity against various bacteria.	[94]
Strawberry	AgNPs@CS	In situ reduction in AgNO_3_ on cellulose using hydroxyl groups; sponge form.	AgNPs: 6.84–54.78 nm. AgNPs uniformly distributed; sponge elastic, 16.8% unrecoverable deformation after 1000 cycles; high biocompatibility.	Extended shelf life to 12 days; protection from both microbial invasion and physical damage under vibration stress.	[95]
Strawberry	LDPE + CNC + Glycerol + Active Formulation (EO + AgNPs) ± γ-irradiation	AgNPs (AGPPH) encapsulated with cinnamon EO; hot-pressed into LDPE blend.	AgNPs: 3–35 nm; CNC-reinforced LDPE; color and mechanical properties altered; stable WVP.	Decay and weight loss reduced by 94% at 12 days; total phenols (952→1711 mg/kg), anthocyanin (185→287 mg/kg); firmness and microbial load preserved.	[96]
Telemea cheese	Alginate film with AgNPs and lemongrass essential oil	Chemical reduction with AgNO_3_ and PVP.	AgNPs: 5–25 nm, spherical, poly/mono-crystalline, and enhanced opacity, thermal, and water vapor properties.	14-day preservation of the cheese, reduced microbial load, maintained softness and surface texture.	[97]
Banana	Green synthesis AgNPs from eucalyptus leaf extract (ELE)	Mixing ELE with AgNO_3_.	AgNP: <100 nm, spherical, high stability.	Extended shelf life of the banana up to 32 days, reduced ethylene production, decay, and weight loss.	[98]
Turkey breast	Z-gAgNPs (Zein + green fabricated AgNPs)	Mixing of green AgNPs synthesized from green tea extract with zein and chemically synthesized AgNPs with NaBH_4_.	Size of chemically synthesized AgNPs: 43.4 ± 21.1 nm. Size of green-synthesized AgNPs: 60.78 nm. Green AgNPs: smaller, spherical, higher stability; Chem. AgNPs: larger, less uniform.	Z-gAgNPs film best preserved quality over 12 days (TVB-N, pH, microbial load); superior to synthetic AgNPs film.	[99]
Papaya	HPMC-based coating	HPMC-AgNP films were prepared by casting and air drying.	AgNPs: 20–100 nm, influenced thickness, moisture content, crystalline structure.	Film inhibited fungal growth, maintained fruit quality, and delayed ripening, extending papaya shelf life significantly.	[100]
Strawberry	PLA film with MPE/AgNPs	PLA solution was cast with MPE/AgNPs after ultrasonication-assisted mixing.	AgNPs: 2.5–6.5 nm, spherical, high stability, improved barrier and mechanical properties.	Strawberries stayed fresh for seven days, extending shelf life four days, and showed high antibacterial activity.	[101]
Blueberry	C-Ag@PVA/CS	Reduction with trisodium citrate onto biochar; added to PVA/CS matrix	Thermally stable, hydrophobic, effective at 3% concentration.	Reduced weight loss and acid degradation, delayed spoilage, effective for blueberry preservation.	[102]
Strawberry	PCP-AgNPs/CS	PCP-AgNPs were incorporated into CS film via solvent casting.	Spherical AgNPs (6.79 nm) stabilized by PCP; film showed improved mechanical strength, thermal stability, and uniform morphology.	Extended strawberry shelf life by 6 days; strong antibacterial activity against various bacteria strains.	[103]
Chicken meat	Starch + PBAT + Glycerol + Lyophilized Bio-AgNPs	Lyophilized bio-AgNPs were manually mixed into film matrix and processed via extrusion into films.	Bio-AgNPs were spherical (81.25 nm, −36.4 mV); films were biodegradable, flexible, thermoplastic, and extrusion-molded.	Films inhibited various bacteria species, extending chicken shelf life up to 10 days.	[104]
Fresh milk	PVA-CNC-AgNPs-CJPE	AgNO_3_ reduced by CJPE, incorporated into PVA-CNC via solvent casting.	Uniform spherical AgNPs; improved UV-blocking, antioxidant, thermal, barrier and mechanical properties.	Extended milk shelf life for 14 days; strong antibacterial activity against various pathogens.	[105]
-	*Thymus vulgaris* extract + AgNPs (TSNPs)	AgNP was obtained by biosynthesis by mixing the AgNO_3_ solution with the plant extract and heating.	AgNPs are 20–40 nm, spherical, brown; show high antioxidant and antimicrobial stability with plant-based surface functionalization.	TSNPs have antibacterial activity against various microorganisms. Also provided DPPH radical scavenging activity.	[106]
Strawberries and chicken breast	CS-Ca-Ag	AgNPs were biosynthesized using *C. roseus* extract and mixed into CS film via solvent casting.	Uniform AgNPs, improved Young’s modulus, WVP, UV blocking, and good thermal stability.	For strawberries: Delayed dehydration and mold growth; preserved firmness, acidity, vitamin C, and color.	[107]
				For chicken breast: Reduced pH rise and MetMb formation; maintained weight and color; slowed microbial spoilage.	
Strawberry	CMC-CNC@AgNPs	AgNPs were immobilized onto CNC and dispersed in the CMC matrix.	AgNPs: 10–20 nm, spherical, well dispersed on CNC. Coated paper showed higher tensile strength, good thermal stability.	Delayed microbial growth, reduced weight loss, retained vitamin C and acidity, slowed TSS degradation; shelf life extended to 7 days.	[108]
-	AgNP incorporated nanocellulose (NC)-Arabinoxylan Acetate (AXAc).	Synthesized AgNPs blended into film-forming emulsions.	AgNPs: 40–70 nm; NC: −34.5 mV zeta; films showed good thermal and barrier traits.	Films showed strong antimicrobial activity against various bacteria, enhancing shelf life.	[109]
-	CS/rGO@AgNPs (Graphene Oxide: GO)	AgNPs immobilized on rGO, then embedded between CH layers forming a film.	Uniform AgNPs (15 ± 5 nm) on rGO; films showed high tensile strength and UV-blocking ability.	Demonstrated excellent antibacterial activity against various bacteria. Exhibited low cytotoxicity.	[110]
Citrus	CMCS@COF-AgNP	AgNPs immobilized into (Covalent Organic Frameworks) COFs via in situ reduction, and then incorporated into carboxymethyl CS (CMCS) films via casting.	AgNP: 46.22 ± 0.97, spherical, uniform dispersion; improved film tensile strength, opacity, WVP, solubility, and swelling.	Reduced weight loss, pH shift, and vitamin C degradation; extended citrus shelf life; excellent antibacterial activity against various bacteria.	[111]
Apple	CG/PVA/AgNPs	AgNPs incorporated into the CG/PVA film via aqueous blending.	AgNPs dispersed in water with CG and PVA, and cast into films via solution casting.	Reduced weight loss, delayed browning, lower PPO activity, and microbial load in samples.	[112]
Bananas	PVA/CNCd/Ag	AgNPs incorporated into the PVA matrix through solution casting.	AgNPs were spherical (~100 nm), uniformly distributed; film showed improved mechanical strength, thermal stability, and UV barrier properties.	PVA/CNCd/Ag film extended banana shelf life to 14 days, reducing decay and maintaining appearance.	[113]
Minced beef	AgPVA nanofibers	AgNPs were blended into a PVA solution and electrospun into nanofibers.	AgNPs were spherical, showed face-centered cubic (FCC) structure; nanofibers were 166–186 nm in diameter with smooth surface and improved crystallinity, enhancing structural stability.	Reduced lipid oxidation, better color retention, and lower microbial growth during 9-day cold storage.	[114]
Chilled beef	POHS/P/BA/AgNPs	Commercial AgNPs were directly mixed with butterfly pea (BA) and polymer solution, then ultrasonicated.	AgNPs were spherical (15–90 nm); films showed enhanced tensile strength, UV-blocking, and thermal stability.	Films visually indicated beef freshness and extended shelf life by ~3 days by inhibiting microbial growth and oxidation.	[87]
Green grape, cherry tomato and mushroom	G-AgNP	AgNPs were incorporated into starch coatings applied on paper surfaces.	AgNPs were spherical (5–20 nm, average 19 nm); improved elongation at break (8.34%), tensile strength, and WVP.	Reduced weight loss in grapes (6.77%) and tomatoes (8.59%); shelf-life extension observed also for mushrooms; strong antimicrobial activity against *E. coli*; low Ag^+^ migration.	[115]
Gouda (rennet-curd) and quark (acid-curd) cheese	Furcelleran (FUR) + AgNPs	AgNO_3_ reduced with xylose in furcellaran solution, dried into film.	AgNPs: 5–20 nm; film transparent; and showed high water vapor permeability and moderate tensile strength.	Reduced yeast and mold in both cheeses, improving microbial stability during 2-week (quark) and 4-week (gouda) storage; silver migrated more in quark cheese and organoleptic quality slightly declined.	[116]
Meat	P70-CH30-AgNLs	AgNPs were blended into the PVA/CH electrospinning solution.	AgNPs averaged 80 ± 11 nm; nanofibers ~196 nm diameter; hydrophobicity improved; nanolayers were thin.	Extended meat shelf life by 7 days; inhibited *E. coli and L. monocytogenes*; reduced microbial growth and odor.	[117]
Red grape	CT/CV/V/CMC/Gin-AgNPs	Gin-AgNPs were mixed into the film-forming solution.	AgNPs enhanced UV-blocking, thermal stability, and mechanical strength, producing flexible, moderately thick films with improved durability.	Maintained grape freshness for 21 days; reduced dehydration and microbial spoilage; >50% biodegradation.	[118]
Tomato	SS-AgNP	SS-AgNPs agar solution to create a homogeneous coating material.	AgNPs were 6–34 nm, mostly polydispersed; films showed good thermal and colloidal stability, and high crystallinity.	SS-AgNP coating reduced tomato weight loss to 15.5% and decay to 6% after 18 days, significantly enhancing shelf life compared to controls.	[119]
Raw cow milk	Paper pad coated with CT-AgNPs	AgNPs directly drop-cast onto paper substrates to form the sensor.	AgNPs were spherical, ~16 nm in size; they showed strong LSPR and good stability against aggregation.	The sensor detected H_2_O_2_ in milk as low as 1.5 ppm, with a detection limit of 8.46 ppm and 92% recovery.	[120]
Salmon fillet	FUR-HGEL/FUR + CAPS-CHIT + AgNPs-YM/FUR + CUR + MMT/FUR (Four layer films tested)	AgNPs incorporated into the second furcellaran layer of the multilayer film during casting.	AgNPs were nanoscale; films showed improved antioxidant activity but slightly reduced mechanical and thermal stability.	Despite no in vitro antimicrobial effect, due to limited release, the films reduced microbial growth in salmon and extended shelf life by 3 days, but failed to prevent lipid oxidation.	[121]
Goat meat	Chi + Alg + NC	AgNPs incorporated into lemongrass nanoemulsion by sonication.	AgNPs were approximately 10 nm in size and spherical in shape. The film was 22.5 ± 1.44 μm thick, smooth, and thermally stable with improved mechanical strength and hydrophilicity.	The film maintained meat color and reduced microbial count below 7 log CFU/g for 7 days, with 54% reduced weight loss compared to control.	[122]
Beef	AG-PAE-TA//C-OPE/TGNP-TA-AgNPs	AgNPs were incorporated into the film via mixing with carrageenan before casting into the agar layer.	AgNPs were spherical, ~50 nm in size; films showed UV-blocking, hydrophobicity, and enhanced tensile strength.	The film extended beef shelf life for 1 day, delayed microbial growth, and enabled freshness monitoring via pH-sensitive color change and smartphone RGB analysis app.	[123]
Rainbow trout fillet	CP-BRPE-gnAg	AgNPs were incorporated into film-forming solutions before casting.	AgNPs were spherical with a diameter of ~51 nm; films showed improved tensile strength, flexibility, and reduced water vapor permeability.	The composite film reduced microbial growth and preserved trout fillets for 12 days, with reduced pH, TVB-N, TBARS, and visible spoilage indicators.	[124]

## Data Availability

No new data were created or analyzed in this study. Data sharing is not applicable to this article.
